# ARMC1 partitions between distinct complexes and assembles MIRO with MTFR to control mitochondrial distribution

**DOI:** 10.1126/sciadv.adu5091

**Published:** 2025-04-09

**Authors:** Michael J. McKenna, Felix Kraus, João P.L. Coelho, Muskaan Vasandani, Jiuchun Zhang, Benjamin M. Adams, Joao A. Paulo, J. Wade Harper, Sichen Shao

**Affiliations:** ^1^ Department of Cell Biology, Harvard Medical School, 240 Longwood Ave., Boston, MA 02115, USA.; ^2^ Howard Hughes Medical Institute, Boston, MA 02115, USA.

## Abstract

Maintaining an optimal mitochondrial distribution is critical to ensure an adequate supply of energy and metabolites to support important cellular functions. How cells balance dynamic mitochondrial processes to achieve homeostasis is incompletely understood. Here, we show that ARMC1 partitioning between distinct mitochondrial protein complexes is a key determinant of mitochondrial distribution. In one complex, the mitochondrial trafficking adaptor MIRO recruits ARMC1, which mediates the assembly of a mitochondrial fission regulator (MTFR). MTFR stability depends on ARMC1, and MIRO-MTFR complexes specifically antagonize retrograde mitochondrial movement. In another complex, DNAJC11 facilitates ARMC1 release from mitochondria. Disrupting MIRO-MTFR assembly fails to rescue aberrant mitochondrial distributions clustered in the perinuclear area observed with ARMC1 deletion, while disrupting ARMC1 interaction with DNAJC11 leads to excessive mitochondrially localized ARMC1 and distinct mitochondrial defects. Thus, the abundance and trafficking impact of MIRO-MTFR complexes require ARMC1, whose mito-cytoplasmic shuttling balanced by DNAJC11 tunes steady-state mitochondrial distributions.

## Teaser

ARMC1 partitions between distinct outer mitochondrial membrane complexes and the cytosol to control mitochondrial distribution.

## INTRODUCTION

Establishing and maintaining a proper distribution of mitochondria, especially in polarized cells such as neurons that have functionally important extensions, is essential to provide an adequate supply of energy and metabolites in a spatially defined manner (*1*, *2*). This requires molecular mechanisms mediated by incompletely characterized protein complexes at the surface of mitochondria to actively balance anterograde and retrograde mitochondrial trafficking. Mechanisms that determine mitochondrial size, number, and morphology also affect the efficiency of mitochondrial trafficking and the appearance of cellular mitochondrial networks. Although aberrant mitochondrial distributions are associated with many aging, metabolic, and neurodegenerative disorders (*1*, *3*), it remains unclear how protein complexes at the outer mitochondrial membrane (OMM) control these dynamic processes.

The best-studied mitochondrial trafficking adaptors are the tail-anchored OMM proteins, MIRO1 and MIRO2 (also known as RHOT1 and RHOT2) (*4*–*6*). We refer to the two human paralogs, which are approximately 60% similar at the sequence level, as MIRO in contexts where they are interchangeable. MIRO interacts with cytoskeletal motor proteins directly or through additional adaptors, such as TRAK1 and TRAK2 (paralogs that we refer to as TRAK when they are interchangeable), that can bind kinesin or dynein to mediate anterograde or retrograde trafficking, respectively (*7*–*13*). While vertebrates have multiple paralogs of these trafficking adaptors, *Drosophila* have only one MIRO (known as dMiro) and one TRAK (known as Milton), and yeast only have a MIRO homolog, Gem1p, and no TRAK (*6*, *12*, *14*–*16*). The expanded collection of mitochondrial trafficking factors in vertebrates likely reflects the increased diversity and complexity of their cells. The mechanisms that regulate interactions with these trafficking adaptors to maintain steady-state mitochondrial distributions are incompletely defined.

There are numerous reports of other proteins that influence mitochondrial distribution (*17*–*19*), but how these proteins function with known trafficking factors such as MIRO or TRAK is poorly understood. One such factor is Armadillo repeat containing protein 1 (ARMC1), an abundant and ubiquitously expressed protein in vertebrates. ARMC1 is dual-localized between the cytosol and the mitochondrial surface and is described to affect mitochondrial distribution through unknown mechanisms (*17*, *20*, *21*). In this study, we report that ARMC1 directly interacts with MIRO and partitions between distinct OMM protein complexes, which is a critical determinant of mitochondrial distribution. Unbiased proteomics and biochemical approaches reveal that ARMC1 mediates MIRO assembly with a mitochondrial fission regulator (MTFR1, MTFR2, or MTFR1L; paralogs collectively referred to as MTFR). MTFR stability depends on ARMC1-mediated complex assembly, which specifically antagonizes excessive retrograde mitochondrial movement through competitive binding with TRAK2. Thus, the abundance of ARMC1-MIRO-MTFR complexes must be controlled to obtain optimal mitochondrial distribution. This is achieved through ARMC1 partitioning into a distinct complex with DNAJC11, which promotes ARMC1 release from mitochondria into the cytosol. Our findings define the spatial partitioning of ARMC1 as a central mechanism for balancing the abundance of specific MIRO complexes needed to maintain steady-state mitochondrial distributions.

## RESULTS

### The ARMC1 CTD mediates mitochondrial localization

ARMC1 contains an N-terminal helical domain with an Armadillo motif, a heavy metal-associated–like (HMAL) domain that lacks cysteines in the positions required for metal coordination, and a short, conserved C-terminal domain (CTD) (fig. S1A). A prior study showed that C-terminally tagged ARMC1 fails to localize to mitochondria (*17*). However, a specific role for the CTD in ARMC1 localization has not been demonstrated.

**Fig. 1.**
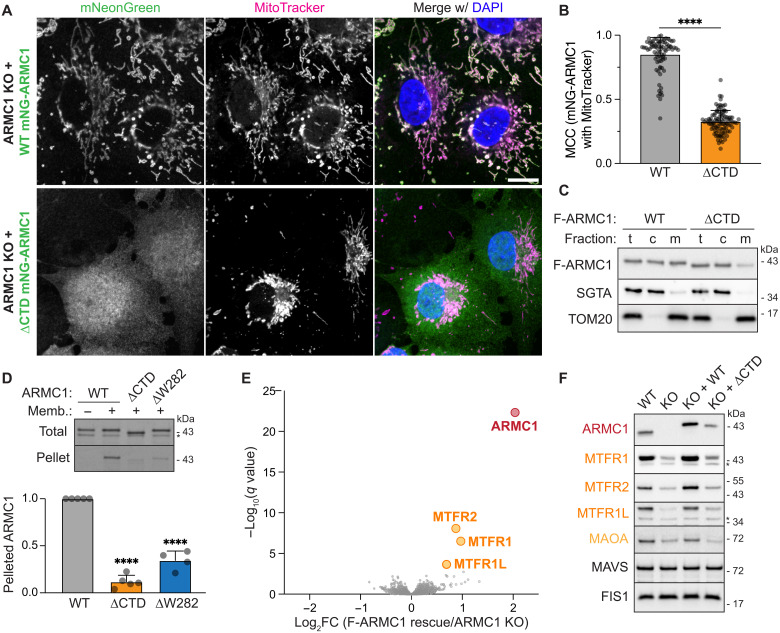
Mitochondrial ARMC1 stabilizes MTFRs. (**A**) ARMC1 CTD is required for mitochondrial localization. Fluorescence microscopy of fixed ARMC1 KO COS7 cells stably re-expressing near-endogenous levels of WT mNG-ARMC1 or mNG-ARMC1 lacking 15 C-terminal residues (ΔCTD), showing mNG-ARMC1 (green), MitoTracker CMXRos (magenta), and nuclei [4′,6-diamidino-2-phenylindole (DAPI), blue]. Scale bar, 15 μm. (**B**) Manders’ colocalization coefficients (MCCs; mean + SD and measurements for *n* = 82 WT and *n* = 100 ΔCTD cells) of the mNG-ARMC1 variants with MitoTracker CMXRos as in (A). ****P < 0.0001. (**C**) Immunoblotting of ARMC1 KO Flp-In 293 T-REx cells re-expressing WT or ΔCTD F-ARMC1 before (t, total) or after fractionation into cytosolic (c) and membrane (m) fractions for F-ARMC1, the cytosolic protein SGTA, and the mitochondrial protein TOM20. (**D**) Radiolabeled ARMC1 variants synthesized in an in vitro mammalian translation system were incubated without or with organelles (memb.) isolated from Expi293F cells and analyzed before (total) or after centrifugation to pellet the membrane fraction by SDS-PAGE and autoradiography (top). The ratio of the F-ARMC1 variant recovered in the pellet fraction relative to WT F-ARMC1 was quantified for four to five independent replicates (bottom). Shown are mean + SD and individual measurements. ****P < 0.0001. (**E**) ARMC1 stabilizes MTFRs. Volcano plot of multiplexed proteomics data showing fold change (FC) of protein levels in ARMC1 KO Flp-In 293 T-REx cells with or without F-ARMC1 rescue. (**F**) SDS-PAGE and immunoblotting of WT or ARMC1 KO Flp-In 293 T-REx cells without or with re-expression of WT or ΔCTD F-ARMC1.

To study the determinants of ARMC1 localization, we generated rescue cell lines, in which we stably complemented ARMC1 knockout (KO) cells with N-terminally tagged ARMC1. We reasoned that this complementation approach would allow us to study the localization and impact of ARMC1 mutants in isogenic backgrounds without possible interference from endogenous ARMC1. Complementing ARMC1 KO COS7 cells with mNeonGreen (mNG)–tagged ARMC1 by stable expression through lentiviral transduction and fluorescence-activated cell sorting for near-endogenous expression levels (fig. S1B) or by transient overexpression (fig. S1C) showed clear colocalization of wild-type (WT) mNG-ARMC1 with mitochondria (Fig. 1, A and B, and fig. S1C). Removing the CTD (residues 268 to 282; ΔCTD) abolished mNG-ARMC1 localization to mitochondria (Fig. 1, A and B, and fig. S1C) and resulted in abnormal mitochondrial distributions (discussed below).

We also rescued ARMC1 KO Flp-In HeLa or 293 T-REx cells with Flag-tagged ARMC1 (F-ARMC1) integrated behind a doxycycline-inducible promoter at the Flp-In locus. We used these cell lines to further confirm the dual cytosolic and mitochondrial localization of WT F-ARMC1 using both immunofluorescence (fig. S1, D and E) and biochemical fractionations to separate cytosolic and membrane-associated contents (Fig. 1C). As in COS7 cells, removing the CTD impaired F-ARMC1 mitochondrial association in both cell types (Fig. 1C and fig. S1, D and E). Thus, the ARMC1 CTD is necessary for mitochondrial localization.

We observed the same CTD dependence in cell-free reconstitutions of ARMC1 targeting to organelles (Fig. 1D). In these assays, we incubated radiolabeled ARMC1 variants synthesized in a mammalian in vitro translation system with crude organelles isolated from Expi293F cells (*22*, *23*). Repelleting the membrane fraction after the incubation period revealed that WT but not ΔCTD ARMC1 specifically associated with the organelles. Notably, removing only the C-terminal tryptophan (W282) was sufficient to substantially disrupt the membrane association of F-ARMC1 (Fig. 1D). However, appending the ARMC1 CTD to mNG was insufficient to confer mitochondrial localization (fig. S1F), suggesting that additional ARMC1 interactions stabilize its mitochondrial association.

### ARMC1 stabilizes MTFRs

To assess the molecular impact of ARMC1 in an unbiased manner, we profiled the proteomes of ARMC1 KO Flp-In 293 or HeLa T-REx cells rescued with WT or ΔCTD F-ARMC1 using multiplexed mass spectrometry through tandem mass tagging (TMT-MS) (Fig. 1E, fig. S2, A and B, and tables S1 and S2). These datasets revealed that the absence of ARMC1 resulted in specific and significant decreases in the levels of MTFR1, MTFR2, and MTFR1L (Fig. 1, E and F, and fig. S2A). These MTFR paralogs (fig. S3) are peripheral mitochondrial proteins that have been reported to impact mitochondrial morphology through unclear mechanisms (*24*–*26*). A link between MTFRs and ARMC1 has not been previously described. Re-expressing WT but not ΔCTD F-ARMC1 specifically rescued MTFR levels (Fig. 1F and fig. S2B). Thus, MTFR levels depend on ARMC1 that can localize to mitochondria.

We observed that MTFR down-regulation occurs through degradation via the ubiquitin-proteasome system, as inhibitors of proteasomes or the E1 ubiquitin-activating enzyme selectively stabilized MTFR (fig. S2, C and D). Furthermore, the population of MTFR stabilized by proteasome inhibition in ARMC1 KO cells is mostly cytosolic (fig. S2E). In Flp-In 293 T-REx cells, the levels of the OMM tail-anchored protein monoamine oxidase A (MAOA) were also dependent on ARMC1. However, unlike MTFRs, MAOA stability was not ARMC1 dependent in Flp-In HeLa T-REx cells, and we did not observe significant ARMC1-dependent changes in the levels of other proteins across different cell types (Fig. 1, E and F; fig. S2A; and tables S1 and S2). These results reveal that ARMC1 uniquely stabilizes MTFRs and facilitates their localization to mitochondria.

### ARMC1 partitions into distinct mitochondrial complexes

**Fig. 2.**
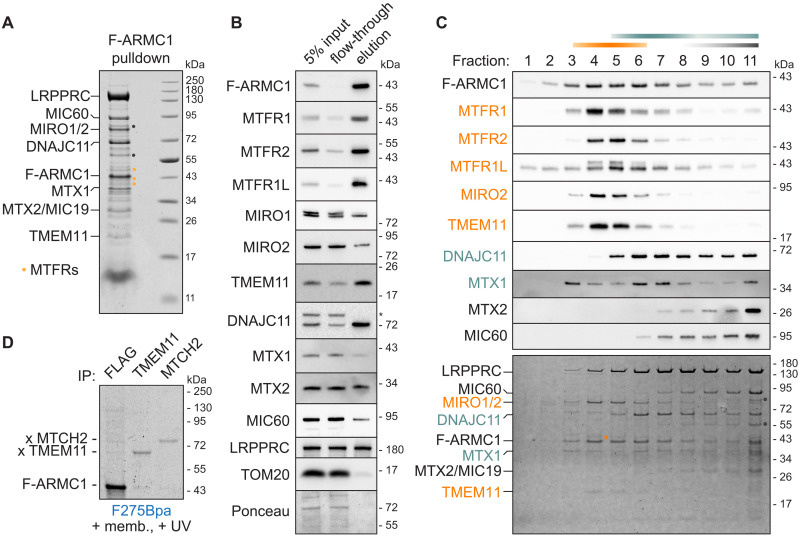
ARMC1 partitions into distinct mitochondrial complexes. (**A**) SDS-PAGE and Coomassie staining of pulldowns (PDs) of F-ARMC1 stably expressed in ARMC1 KO Flp-In 293 T-REx cells, with proteins identified by mass spectrometry indicated. Orange dots, MTFRs. Gray dots, mitochondrial intermembrane bridging (MIB) complex proteins. (**B**) Immunoblotting of the solubilized membrane fraction (input), flow-through, and elution of F-ARMC1 affinity purification as in (A). (**C**) SDS-PAGE and immunoblotting (top) or Coomassie staining (bottom) of F-ARMC1 PD as in (A) size fractionated over a 10 to 30% sucrose gradient. Orange, teal, and gray lines indicate distinct complexes. (**D**) TMEM11 and MTCH2 bind the ARMC1 CTD. In vitro translation reactions of radiolabeled F-ARMC1 with the UV-activated Bpa probe at position 275 (F275Bpa) in the CTD incubated with organelles isolated from Expi293F cells were UV irradiated, immunoprecipitated (IP) for crosslinks to MTCH2 and TMEM11, and assayed by SDS-PAGE and autoradiography.

To identify mitochondrial interacting partners of ARMC1, we affinity purified F-ARMC1 from solubilized membrane fractions of Flp-In 293 T-REx rescue cells (Fig. 2, A and B, and table S3). Of the copurified proteins identified by mass spectrometry, we assigned those localized at the mitochondrial surface (e.g., OMM or peripheral proteins) as candidates most likely to directly bind ARMC1. Among these, we identified the two MIRO paralogs, the multispanning OMM protein TMEM11 (*27*), and all three MTFRs as previously unappreciated F-ARMC1 interactors. Although MTFRs were substoichiometric among F-ARMC1 interactors (Fig. 2A), they are also several-fold less abundant than ARMC1 in cells (fig. S4A) (*20*, *21*), and each MTFR was substantially depleted from the flow-through of the F-ARMC1 pulldown (Fig. 2B). Considered together with our finding that ARMC1 stabilizes MTFRs (Fig. 1, E and F, and fig. S2A), these results suggest that ARMC1 is a major interactor of MTFRs.

Consistent with prior work (*17*), other OMM proteins identified to copurify with F-ARMC1 included DNAJC11, metaxin 1 (MTX1), and metaxin 2 (MTX2), which are characterized as components of the mitochondrial intermembrane space bridging (MIB) complex involved in mitochondrial cristae organization (*28*–*31*). The MIB complex also includes the mitochondrial contact site and cristae organizing system (MICOS), whose subunits reside at the inner mitochondrial membrane and in the intermembrane space (fig. S4B) (*28*, *29*). Other MICOS components, such as MIC60 and MIC19, were also identified in the affinity purification (Fig. 2, A and B, and table S3). However, because ARMC1 resides on the cytosolic side of the OMM, these factors probably interact with ARMC1 indirectly through an intermediate binding partner, such as an MIB subunit embedded in the OMM. The same consideration applies to LRPPRC (leucine-rich pentatricopeptide repeat containing protein), which also pulled down with ARMC1 but is best characterized to regulate translation in the mitochondrial matrix (*32*–*34*) (fig. S4B). In addition, unlike the MTFRs or DNAJC11, LRPPRC and MICOS subunits were not noticeably depleted in the flow-through after F-ARMC1 purification (Fig. 2B), suggesting that only a small proportion of these factors are part of complexes that contain ARMC1. We therefore prioritized further analyses on potential direct binding partners of ARMC1 at the OMM.

Size fractionations of F-ARMC1 affinity purifications revealed that ARMC1 partitions between at least two distinct OMM complexes (Fig. 2C, indicated in orange and teal). One complex comprises MIRO, MTFR, and TMEM11. A heavier complex contains DNAJC11 and MTX1, but not MTX2 or other MIB/MICOS components, which were only found in the heaviest fractions of the F-ARMC1 purifications and of solubilized mitochondria (Fig. 2C and fig. S4C) (*35*). DNAJC11 is an essential OMM protein predicted to have both β barrel and α-helical transmembrane domains as well as an N-terminal cytosolic Hsp70-recruiting J domain, while MTX1 is N-terminally anchored in the OMM. The sucrose gradient migration patterns of the proteins that copurify with F-ARMC1 generally match their migration patterns in size fractionations of freshly solubilized cellular membranes (fig. S4C), suggesting that the complexes copurified with F-ARMC1 largely represent their native states.

To test whether these ARMC1-containing OMM complexes are indeed physically distinct, we knocked down components of each complex and then performed F-ARMC1 pulldowns (fig. S4D). Knocking down MIRO1 and MIRO2 selectively impaired the association of F-ARMC1 with MTFR and TMEM11 but had no impact on ARMC1 association with DNAJC11 and MTX1. We observed similar results when we knocked down the three MTFRs and to a lesser extent, TMEM11. Reciprocally, knocking down DNAJC11 selectively impaired the copurification of both DNAJC11 and MTX1 with F-ARMC1. Thus, ARMC1 can reside independently in compositionally distinct complexes at the surface of mitochondria.

### TMEM11 and MTCH2 are ARMC1 CTD receptors

Because the ARMC1 CTD is critical for mitochondrial localization (Fig. 1, A to D), we next identified direct interactors of the CTD using site-specific photocrosslinking. To do this, we incorporated the ultraviolet (UV)–activated probe *p*-benzoyl-l-phenylalanine (Bpa) in place of phenylalanine at position 275 within the CTD of radiolabeled ARMC1 [ARMC1(F275Bpa)] using in vitro translation reactions containing an amber stop codon suppression system (*36*). The UV irradiation of ARMC1(F275Bpa) incubated with cellular membranes revealed several CTD-specific crosslinks (fig. S5A). Targeted immunoprecipitations identified TMEM11 and MTCH2 (mitochondrial carrier 2, also known as SLC25A50) as two crosslinking partners of the ARMC1 CTD (Fig. 2D and fig. S5A).

We tested TMEM11 as a crosslinking candidate because of its identification as an ARMC1 interactor (Fig. 2, A to C) and because its notably small size (~20 kDa) was consistent with a prominent crosslink with ARMC1(F275Bpa). MTCH2 is a modified solute carrier at the OMM that is linked to a wide range of functions, including lipid transport (*37*, *38*), mitochondrial fusion (*39*–*41*), apoptosis (*42*–*44*), and OMM protein insertion (*45*). The observation that MTCH2 displays strong genetic interactions with several ARMC1 interactors, particularly DNAJC11 and TMEM11 (*46*), as well as its molecular weight prompted us to test MTCH2 as a potential crosslinking partner of ARMC1(F275Bpa). Subsequent targeted analyses detected a specific interaction between MTCH2 and ARMC1 through both site-specific photocrosslinking (Fig. 2D and fig. S5A) and affinity purifications (fig. S4D and table S3). The MTCH2 interaction was specific to the ARMC1 CTD, as radiolabeled ARMC1 with Bpa incorporated at position 47 in the helical domain primarily crosslinked to MTX1 and not MTCH2 (fig. S5A). Notably, replacing the ARMC1 CTD with a transmembrane helix that independently localizes to the OMM selectively bypassed the interaction of F-ARMC1 with both TMEM11 and MTCH2 but not with other ARMC1 interactors (fig. S5B). Thus, TMEM11 and MTCH2 are specific ARMC1 CTD receptors at the OMM.

**Fig. 3.**
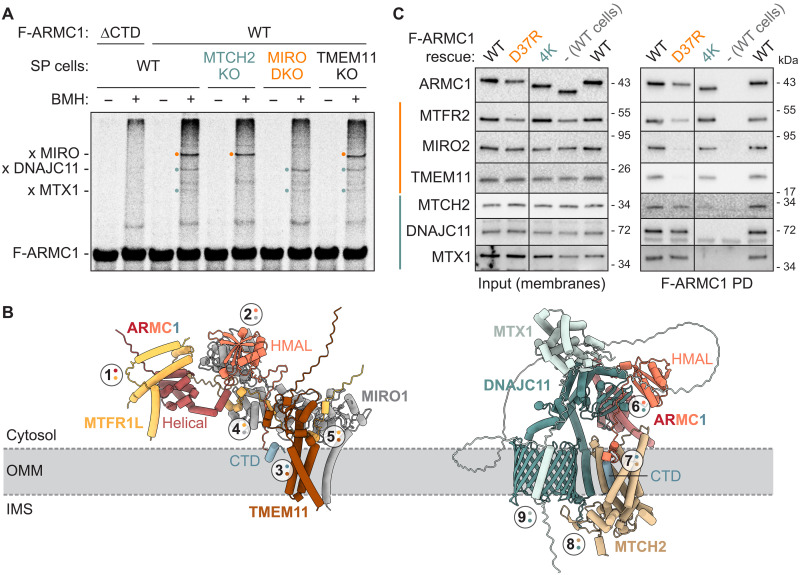
Uncoupling ARMC1 association with distinct mitochondrial complexes. (**A**) MTCH2 is required for ARMC1 association with DNAJC11. Chemical crosslinking reactions of ΔCTD or WT-radiolabeled F-ARMC1 incubated with WT, MTCH2 KO, MIRO1 and MIRO2 double KO (DKO), or TMEM11 KO semi-permeabilized (SP) cells, analyzed by SDS-PAGE and phosphor imaging. Crosslinks to MIRO (orange dot), DNAJC11, and MTX1 (teal dots) are indicated. (**B**) Alphafold models of the indicated ARMC1 complexes with inferred placement of the OMM and protein topologies. IMS, intermembrane space. Circled numbers note interaction interfaces between two proteins as indicated by colored dots (ARMC1 helical domain, dark red; ARMC1 HMAL domain, salmon; ARMC1 CTD, light blue; MTFR, light orange; MIRO, gray; TMEM11, brown; DNAJC11, dark teal; MTCH2, tan; MTX1, light teal). (**C**) Structure-guided mutations uncouple ARMC1 complex association. Immunoblotting of membrane fractions (input, left) and pulldowns (right) of the indicated F-ARMC1 variants expressed in ARMC1 KO Flp-In 293 T-REx cells. 4K: D239K, E243K, D244K, and E245K.

Size fractionations of F-ARMC1 purifications showed that the specific population of MTCH2 associated with F-ARMC1 comigrates with DNAJC11 (fig. S5C). In addition, MTCH2 depletion disrupted F-ARMC1 association with DNAJC11 and MTX1 but left the ARMC1-MIRO-MTFR-TMEM11 complex intact (fig. S4D). Both site-specific and cysteine-based crosslinks of F-ARMC1 with DNAJC11 and/or MTX1 were also specifically lost with organelles isolated from MTCH2 KO cells (Fig. 3A and fig. S5, D and E). In comparison, knocking out MIRO1 and MIRO2 selectively abolished ARMC1-MIRO crosslinks without affecting ARMC1 crosslinking to DNAJC11 and MTX1 (Fig. 3A). Knocking out TMEM11 did not substantially change the major crosslinks (Fig. 3A), consistent with our affinity purification analyses (fig. S4D). Thus, while TMEM11 is an ARMC1 CTD receptor associated with MIRO and MTFR (Fig. 2, C and D), MTCH2 is an ARMC1 CTD receptor that facilitates ARMC1 interaction with DNAJC11 and MTX1.

### Uncoupling ARMC1 association with different mitochondrial complexes

Alphafold3 predictions revealed plausible and consistent assemblies of the ARMC1-MIRO-MTFR-TMEM11 and the ARMC1-DNAJC11-MTX1-MTCH2 complexes (Fig. 3B and fig. S6) (*47*). The remainder of our study focuses on these two currently uncharacterized assemblies because the Alphafold3 models facilitate molecular-level analyses and because the majority of F-ARMC1 resides in fractions corresponding to these two complexes (Fig. 2C and fig. S4C). Alphafold3 predictions of ARMC1 with MIRO, MTFR, and TMEM11 show conserved direct interactions between the ARMC1 helical domain and MTFR (interaction 1), the ARMC1 HMAL domain and MIRO (interaction 2), the ARMC1 CTD and TMEM11 (interaction 3), MTFR and MIRO (*48*) (interaction 4), and MTFR and TMEM11 (interaction 5) (Fig. 3B and figs. S6A and S7, A to C). These interactions were predicted with both MIRO1 and MIRO2 and with all three MTFRs (figs. S6A and S7A) but are compatible with the assembly of only one MTFR and one MIRO at a time. Models of the ARMC1-DNAJC11-MTX1-MTCH2 complex indicate interactions between the ARMC1 HMAL domain and the cytosolic C-terminal domain of DNAJC11 (interaction 6), the ARMC1 CTD and MTCH2 (interaction 7), DNAJC11 and MTCH2 (interaction 8), and DNAJC11 and MTX1 (interaction 9) (Fig. 3B and figs. S6B and S7, D and E).

The models also indicate that the ARMC1 CTD may directly contact the OMM when bound to TMEM11 or MTCH2 (fig. S7, B to D). On TMEM11, a basic patch at the cytosolic face of the OMM may engage the C-terminal carboxyl group of ARMC1 (fig. S7B). In this position, the CTD of ARMC1 may form an amphipathic helix (fig. S7C) that partially embeds into the OMM. Similarly, unlike canonical solute carriers that have six transmembrane helices, MTCH2 is predicted to have only five, and Alphafold models consistently place the ARMC1 CTD in the position of the “missing” sixth transmembrane helix (fig. S7D). In this position, the CTD also forms an amphipathic helix with the hydrophobic side facing the lipid bilayer of the OMM and the C-terminal carboxyl group interacting with lysine 25 of MTCH2.

These predictions allowed us to use Alphafold-guided mutagenesis to selectively disrupt the assembly of one ARMC1 complex at a time. To disrupt the ARMC1-MIRO-MTFR-TMEM11 complex, we used an ARMC1 variant predicted to impair the interaction with MTFR. The Alphafold models showed a common binding interface between the ARMC1 helical domain and two conserved MTFR helices that is stabilized by electrostatic interactions involving D27, D37, and D49 of ARMC1 (interaction 1; Fig. 3B and figs. S3, S6A, and S7A). Introducing a single-point mutation, D37R, at this interface of F-ARMC1 disrupted the copurification of MTFR, MIRO, and TMEM11 without impairing the association of DNACJ11 or MTX1 (Fig. 3C).

To disrupt the interaction between ARMC1 and DNAJC11, we reversed the charges of four residues (D239, E243, D244, and E245) in an acidic linker of ARMC1 that appears to specifically interact with a basic patch on DNAJC11 (fig. S7E). This “4K” mutant selectively disrupted F-ARMC1 interaction with DNAJC11, MTX1, and MTCH2, but not with MIRO, MTFR, or TMEM11 (Fig. 3C). Thus, ARMC1 partitions between at least two distinct mitochondrial complexes, and we have identified unique mutations to specifically uncouple the association of ARMC1 with each complex.

### ARMC1 mediates the assembly of one MTFR with one MIRO

The separation-of-function mutants of ARMC1 identified above have distinct impacts on MTFR stability. D37R F-ARMC1 failed to rescue MTFR levels (fig. S8, A and B), even when overexpressed (fig. S8B, lane 8), while 4K F-ARMC1 rescued MTFR levels to a similar extent as WT F-ARMC1 (fig. S8B, lanes 9 to 11). In addition, overexpressing any MTFR destabilized endogenous MTFRs (fig. S8C), and introducing two charge-reversal mutations (A-2D) into MTFR predicted to disrupt interaction with ARMC1 abolished this effect (fig. S8C). Thus, in support of our quantitative proteomics data (Fig. 1E), an interaction between ARMC1 and MTFR is important for MTFR stability.

Pulldowns of Strep-tagged MTFR2 (S-MTFR2) copurified ARMC1, MIRO, and TMEM11, but not DNAJC11, MTX1, or either of the other two MTFRs (fig. S9, A and B). This is consistent with the absence of MTFR in the ARMC1-DNAJC11 complex and the prediction that only one MTFR assembles with ARMC1 and MIRO at a time (Fig. 3B). In comparison, S-MTFR2 expressed in ARMC1 KO cells was impaired in pulling down MIRO, as was A-2D S-MTFR2, which contains mutations that disrupt ARMC1 binding, expressed in WT cells (fig. S9B). These results support a crucial role for ARMC1 in assembling MTFR with MIRO.

Unexpectedly, the analysis of the individual MTFR paralogs revealed specificity in their interactions (fig. S9C). While all three MTFRs pulled down MIRO2, only S-MTFR1L pulled down MIRO1. Mirroring this specificity, knocking out MIRO1 destabilized MTFR1L but not MTFR1 or MTFR2 (fig. S9D). Reciprocally, knocking out MIRO2 destabilized MTFR1 and MTFR2 but not MTFR1L. In addition, overexpressing S-MTFR2 but not S-MTFR1 or S-MTFR1L destabilized TMEM11 (fig. S9C). TMEM11 destabilization by S-MTFR2 overexpression was disrupted by the A-2D mutations that disrupt the ARMC1 interaction site (fig. S9B) and by charge reversal mutations of three MTFR2 residues (K29, R35, and R39; T-3D) that are predicted to interact with the cytosolic domain of TMEM11 (fig. S9E). The specific effects of individual MIRO and MTFR paralogs may tune the function of ARMC1-MIRO-MTFR-TMEM11 assemblies (discussed below) under different cellular conditions or in diverse cell types, where these paralogs are differentially expressed and are subject to distinct regulation through posttranslational modifications (*26*, *49*, *50*).

### The spatial balance of ARMC1 affects mitochondrial distribution

**Fig. 4.**
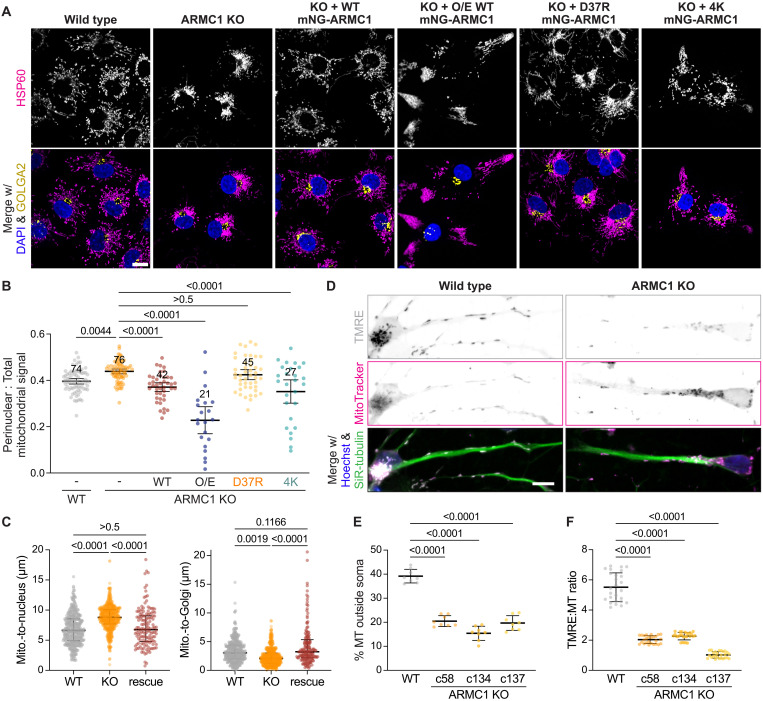
ARMC1 partitioning impacts mitochondrial distribution. (**A**) ARMC1 levels and variants change mitochondrial distribution. Immunofluorescence of WT and ARMC1 KO COS7 cells complemented without or with the indicated mNG-tagged ARMC1 variants, showing the mitochondrial protein HSP60 (magenta), the Golgi marker GOLGA2 (yellow), and nuclei (DAPI, blue). Scale bar, 15 μm. O/E, overexpressed. (**B**) Proportion of HSP60 signal within a dilated nuclear mask of cells as in (A). Shown are the mean, 95% confidence interval, the number of fields of view, and *P* values. (**C**) Distances between the COMs of HSP60 (mito.) to DAPI (left) or GOLGA2 (right) signals in WT (*n* = 329), ARMC1 KO (*n* = 457), or mNG-ARMC1 rescue (*n* = 141) cells as in (A). Shown are median, interquartile range, individual values, and *P* values. (**D**) ARMC1 is important for mitochondrial distribution in iNeurons. Live-cell images of WT or ARMC1 KO iNeurons after 12 days of NGN2-induced differentiation showing polarized mitochondria (TMRE, white and top), total mitochondria (MitoTrackerGreen, magenta), nuclei (Hoechst, blue), and microtubules (SiR-tubulin, green). Scale bar, 10 μm. (**E**) Percentage of MitoTrackerGreen (MT) signal outside of a soma mask to total MT signal in a SiR-tubulin mask of WT and three clonal lines of ARMC1 KO iNeurons after 20 days of differentiation from eight different fields of view each. (**F**) Ratio of TMRE to MT signal of WT and ARMC1 KO iNeurons after 20 days of differentiation from 26 different fields of view each.

We next aimed to connect our biochemical insights to cellular phenotypes. Knocking out ARMC1 severely disrupts mitochondrial distribution (Fig. 4) (*17*). In dividing cells, especially in COS7 cells, deleting ARMC1 caused the distribution of mitochondria to “collapse” into the perinuclear region (Fig. 4, A and B, and fig. S10A). The mitochondrial distributions in ARMC1 KO cells were also more asymmetric, clustered around the Golgi apparatus (Fig. 4A), which generally coincides with the microtubule organizing center. As a result, the distance between the center of mass (COM) of the mitochondrial signal and that of the nuclear signal increased, while the distance between the mitochondrial COM and the Golgi COM decreased upon ARMC1 deletion (Fig. 4C). We show that complementing ARMC1 KO COS7 cells with mNG-tagged ARMC1 at near-endogenous levels can fully rescue these aberrancies of mitochondrial distribution (Fig. 4, A to C, and fig. S10B). Thus, ARMC1 has a specific impact on mitochondrial distribution.

Considering the particular importance of mitochondrial availability in neuronal extensions, we also explored the impact of ARMC1 in neurogenin2 (NGN2)–induced neurons (iNeurons). As in dividing cells, ARMC1 deletion in iNeurons lowered the levels of MTFR1L (fig. S11A), which is specifically up-regulated during iNeuron differentiation, while MTFR1 and MTFR2 are down-regulated (fig. S11B) (*49*). Similar to MTFR1L, MIRO1 is up-regulated during iNeuron differentiation (fig. S11B) (*49*), further supporting a specific link between these paralogs (fig. S9C). Notably, deleting ARMC1 resulted in a notable depletion of mitochondria from iNeuron extensions (Fig. 4, D and E, and fig. S11, C and D). In addition, comparing the ratio of tetramethylrhodamine, ethyl ester, perchlorate (TMRE) labeling, which specifically accumulates in mitochondria with intact membrane potential (*51*), to MitoTrackerGreen labeling of total mitochondria revealed that mitochondria are more depolarized in ARMC1 KO iNeurons (Fig. 4F). These analyses across different cell lines show that ARMC1 is ubiquitously important for mitochondrial distribution and function.

Compared to the impact of knocking out ARMC1, overexpressing mNG-ARMC1 yielded the opposite phenotype, in which mitochondria were found nearly exclusively at the extreme periphery of cells (Fig. 4, A and B, and fig. S10B). This indicates that ARMC1 levels dominantly affect cellular mitochondrial distribution. We did not detect ARMC1 interaction with any known trafficking factors other than MIRO or with any mitochondrial fission or fusion factors other than MTFR (fig. S12A), although mitochondria in both ARMC1 KO COS7 cells and iNeurons displayed fewer fission events (fig. S12B and movie S1). We also observed no changes in the microtubule network in ARMC1 KO iNeurons or COS7 cells (figs. S11C and S12C). These observations suggest that ARMC1 exerts a specific activity on mitochondrial distribution through a currently uncharacterized mechanism, most likely via its interaction with MIRO and MTFR.

Consistent with this idea, complementing ARMC1 KO COS7 cells with D37R mNG-ARMC1, which does not assemble with MIRO and MTFR (Fig. 3C), failed to rescue the aberrant mitochondrial distribution phenotype of deleting ARMC1, as reflected by the persistent accumulation of mitochondria in the perinuclear region (Fig. 4, A and B, and fig. S10B). Complementation with 4K mNG-ARMC1, which can assemble with MIRO and MTFR but not DNAJC11, resulted in distinct aberrancies (Fig. 4A and fig. S10B). On average, 4K ARMC1 re-established mitochondrial localization to the cellular periphery but with heterogeneous cell-to-cell distributions, including more cells with phenotypes resembling ARMC1 overexpression (Fig. 4, A and B). Thus, the spatial partitioning of ARMC1, particularly its ability to assemble with MIRO and MTFR, is critical for cells to achieve optimal mitochondrial distributions.

### ARMC1-MIRO-MTFR assemblies counter retrograde movement

**Fig. 5.**
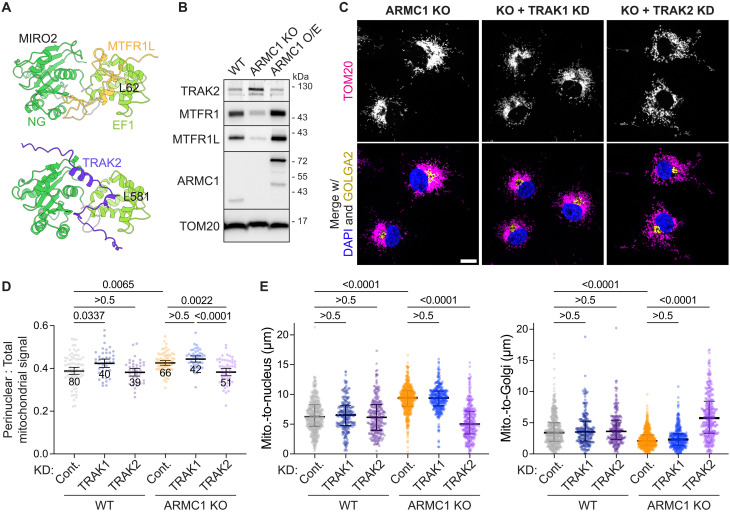
ARMC1-mediated MIRO-MTFR assembly competes with TRAK2 activity. (**A**) MTFR and TRAK engage a common MIRO binding site. Alphafold models of MTFR1L or TRAK2 with MIRO2 predict a common interaction interface with the N-GTPase (NG) and first EF-hand (EF1) domain of MIRO. (**B**) ARMC1 impacts TRAK mitochondrial recruitment. Immunoblotting of membrane fractions of WT and ARMC1 KO COS7 cells without or with overexpression of mNG-tagged ARMC1 shows increased TRAK2 recovery in the absence of ARMC1. (**C**) TRAK2 depletion specifically rescues the ARMC1 KO phenotype. Immunofluorescence showing the mitochondrial protein TOM20 (top or magenta), the Golgi marker GOLGA2 (yellow), and nuclei (DAPI, blue) of ARMC1 KO cells without or with siRNA-mediated knockdown (KD) of TRAK1 or TRAK2. Scale bar, 15 μm. (**D**) Proportion of TOM20 signal within a dilated nuclear mask of WT or ARMC1 KO cells treated with control (cont.) siRNAs or siRNAs against TRAK1 or TRAK2 and imaged as in (C). Shown are the mean, 95% confidence interval, the number of fields of view analyzed, and *P* values. (**E**) Distance between the COMs of TOM20 (mito.) to DAPI (nucleus, left) or GOLGA2 (Golgi, right) signals in cells imaged as in (C). Shown are median, interquartile range, and individual values (WT, *n* = 393, 201, and 208; ARMC1 KO, *n* = 430, 274, and 280 for control, TRAK1, and TRAK2 KD cells, respectively).

We hypothesized that the aberrant mitochondrial distribution phenotypes associated with ARMC1 loss of function and overexpression may result from the ARMC1-mediated assembly of MTFR competing with cytoskeletal adaptors for MIRO engagement. All three MTFRs are predicted to interact with the MIRO EF1 domain (*48*) (interaction 4, Fig. 3B), where F76 of MTFR1 or MTFR2 or L62 of MTFR1L occupies a hydrophobic pocket on MIRO (Fig. 5A and fig. S13A). This binding site on MIRO appears to be a hotspot for functional interactions and is also predicted to engage TRAK (Fig. 5A) (*48*), suggesting competition for MIRO binding.

Supporting this idea, we recovered more TRAK2 with cellular membranes from ARMC1 KO cells, while mNG-ARMC1 overexpression led to less membrane-bound TRAK2 (Fig. 5B). Membrane-associated TRAK2 was also enriched in cells expressing D37R but not 4K mNG-ARMC1 (fig. S13B). This shows that the specific function of ARMC1 in assembling MIRO with MTFR antagonizes TRAK2 recruitment to cellular membranes. We could also directly recapitulate this competition by overexpressing MTFR1L or TRAK2 variants (fig. S13C). Overexpressing MTFR1L resulted in less membrane-associated TRAK2 relative to WT cells. Overexpressing “M-4A” MTFR1L carrying mutations of the “LADI” motif, beginning with L62 predicted to occupy the MIRO EF1 pocket, abolished this competitive effect (Fig. 5A and figs. S3 and S13C). Reciprocally, overexpressing WT TRAK2 but not a variant harboring a single-point mutation (L581A) of the residue predicted to bind the MIRO EF1 pocket reduced MTFR1L membrane association (fig. S13C). Thus, ARMC1-MIRO-MTFR assemblies compete with TRAK2 recruitment to mitochondria.

Strikingly, knocking down TRAK2 rescued the ARMC1 KO phenotype in COS7 cells and produced mitochondrial distributions resembling WT cells, while knocking down TRAK1 had no observable effect (Fig. 5, C to E, and fig. S14, A and B). This effect was specific not only between the TRAK paralogs but also to ARMC1 KO cells, as TRAK depletion had minimal impact on mitochondrial distributions in WT cells (Fig. 5, D and E, and fig. S14C). These observations suggest that TRAK2 actively contributes to the aberrant “collapsed” mitochondrial distribution observed with ARMC1 deletion. This conclusion is supported by reports linking TRAK2 to enhanced retrograde mitochondrial trafficking and other functional differences noted between the TRAK paralogs, particularly in neurons (*8*, *9*, *11*, *13*, *52*). These results indicate that the loss-of-function ARMC1 phenotype in COS7 cells primarily arises from an aberrant gain of TRAK2-driven retrograde mitochondrial movement.

### MIRO and DNAJC11 have opposing effects on ARMC1 localization

Because ARMC1-MIRO-MTFR complexes prevent excessive retrograde mitochondrial movement (Fig. 5), we reasoned that the spatial partitioning of ARMC1 helps to control the abundance, and thus the functional impact, of ARMC1-MIRO-MTFR assemblies (Fig. 4, A and B). In addition, we have shown that ARMC1 partitions not only between mitochondria and the cytosol (Fig. 1, A to C) but also between distinct protein complexes at the OMM (Fig. 2, A to C). Unexpectedly, these distinct OMM complexes also have opposing effects on the mitochondrial localization of ARMC1 itself.

**Fig. 6.**
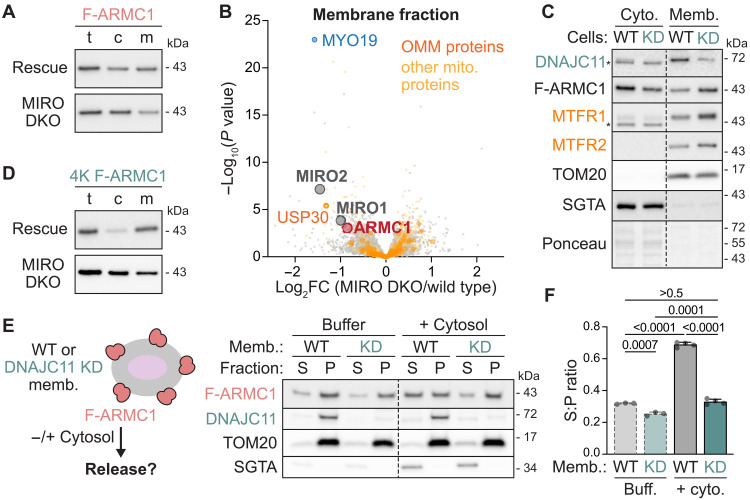
MIRO and DNAJC11 have opposing effects on ARMC1 localization. (**A**) ARMC1 membrane association decreases with MIRO deletion. Immunoblotting of F-ARMC1 Flp-In 293 T-REx rescue cells without or with MIRO1 and MIRO2 double KO (DKO) directly (total, t) or after separation of cytosolic (c) and membrane (m) fractions. (**B**) Volcano plot of multiplexed proteomics data showing the fold-change (FC) of proteins associated with cellular membranes in MIRO1 and MIRO2 DKO relative to WT Flp-In 293 T-REx cells. Individual, OMM, and other mitochondrial proteins are indicated. (**C**) ARMC1 and MTFR membrane association increases with DNAJC11 depletion. Immunoblotting of the cytosolic and membrane fractions of F-ARMC1 rescue cells without or with siRNA-mediated KD of DNAJC11. (**D**) As in (A) but using 4K F-ARMC1 rescue cells without or with MIRO DKO. (**E**) DNAJC11 promotes ARMC1 release from mitochondria. Scheme (left) and immunoblots (right) of F-ARMC1 mitochondrial release assays, in which the membrane fraction (memb.) of F-ARMC1 rescue cells without or with DNAJC11 KD were incubated without or with cytosol and then separated into supernatant (S) and membrane pellet (P) fractions. (**F**) Ratios of radiolabeled F-ARMC1 signal in the supernatant (S) and pellet (P) fractions of release assays in which in vitro synthesized radiolabeled F-ARMC1 was first pre-incubated with membrane fractions (memb.) from cells without or with DNAJC11 KD, which were then re-isolated for release assays in the presence of buffer (buff.) or cytosol as in (E). Shown are means + SD, individual values, and *P* values for three to four independent replicates.

We first noticed that depleting MIRO, but not other ARMC1 interactors, reduced the proportion of F-ARMC1 associated with cellular membranes (Fig. 6A and fig. S4D). To assess this effect in an unbiased manner, we used TMT-MS to profile the proteins that cofractionate with cellular membranes after knocking out MIRO1, MIRO2, or both [double KO (DKO)]. These datasets revealed that endogenous ARMC1 is one of the most significantly depleted proteins from the membrane fraction upon deleting MIRO1 and/or MIRO2 (Fig. 6B; fig. S15, A and B; and table S4).

Deleting MIRO1 and MIRO2 also disrupted the membrane association of other MIRO interactors, such as TRAK2 (which is not detected by TMT-MS) and MYO19, an actin-based motor protein that may facilitate mitochondrial segregation during cell division (Fig. 6B and fig. S15B) (*9*, *10*, *48*, *53*). Notably, while deleting either MIRO paralog reduced ARMC1 membrane association, the recovery of membrane-associated MYO19 was specifically perturbed by knocking out MIRO2 but not MIRO1 (fig. S15, A and C, and table S4). This finding supports prior reports that link MYO19 to MIRO2 (*9*, *54*) and further highlights the functional differences between the MIRO paralogs, similar to our observations that only MTFR1L levels decrease upon MIRO1 deletion (figs. S9, C and D, and S15C). However, unlike MTFRs, which depend on ARMC1 for stability (Fig. 1, E and F, and figs. S2 and S15C), or TRAK2, which has a competitive relationship with ARMC1 (Fig. 5B), the membrane association of MYO19 was not affected by ARMC1 deletion (fig. S15C). This suggests that MYO19 and ARMC1 engage MIRO independently. The membrane recovery of both ARMC1 and MYO19 was rescued by re-expressing Flag-tagged MIRO2 (F-MIRO2) in MIRO2 KO or MIRO DKO cells (fig. S15D and table S5). Collectively, these data support the conclusion that MIRO promotes ARMC1 association with mitochondria.

Unexpectedly, depleting DNAJC11 produced the opposite effect to MIRO depletion. Although knocking down DNAJC11 theoretically removes an ARMC1 binding site from mitochondria, we consistently observed more ARMC1 and MTFR in the membrane fraction of cells depleted of DNAJC11 (Fig. 6C). In addition, 4K F-ARMC1, which still interacts with MIRO but fails to assemble with DNAJC11 (Fig. 3C and fig. S16A), is found almost exclusively at cellular membranes in a MIRO-dependent manner (Fig. 6D). The inability to bind DNAJC11 therefore paradoxically enhances ARMC1 mitochondrial association.

These observations suggest that DNAJC11 promotes ARMC1 release from mitochondria into the cytosol. To probe this hypothesis, we staged in vitro ARMC1 release assays (Fig. 6E and fig. S16B) by incubating membrane fractions isolated from cells expressing F-ARMC1 variants with either buffer or cytosol isolated from WT cells. We then assayed how much F-ARMC1 was released into the supernatant after repelleting the membranes. This revealed that the the addition of cytosol enhanced the release of WT but not 4K F-ARMC1 from mitochondria and that depleting DNAJC11 impaired F-ARMC1 release (Fig. 6E and fig. S16B).

We obtained similar results for F-ARMC1 exogenously added from in vitro translation reactions. In these assays, we incubated equal amounts of radiolabeled F-ARMC1 with organellar membranes from cells with or without DNAJC11 knockdown. These membrane fractions were then re-isolated for release assays with buffer or cytosol as described above (fig. S16C). Quantifying the ratio of radiolabeled F-ARMC1 released into the supernatant to what remained in the pellet showed that significantly more F-ARMC1 is released from WT membranes in the presence of cytosol than in all other conditions (Fig. 6F and fig. S16C). These results support the conclusion that DNAJC11 facilitates ARMC1 removal from mitochondria.

**Fig. 7.**
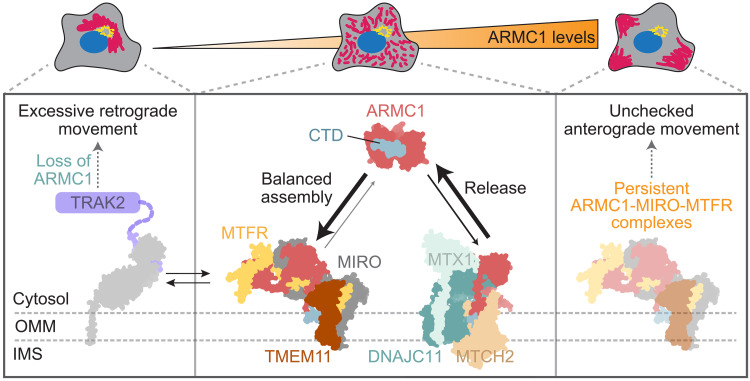
ARMC1 partitioning is a major determinant of mitochondrial distribution. Model (center) of ARMC1 partitioning between the cytosol and distinct mitochondrial complexes, which requires the CTD of ARMC1. ARMC1 mediates the assembly of MIRO with MTFR and TMEM11. This complex is required for MTFR stability and competes with TRAK2 recruitment to mitochondria. ARMC1 can also engage a complex containing MTX1, MTCH2, and DNAJC11, which promotes ARMC1 release from mitochondria. Imbalances in ARMC1 partitioning between these interactors result in aberrant mitochondrial distributions. If ARMC1 fails to assemble with MIRO and MTFR, then too much mitochondrial recruitment of TRAK2 drives aberrantly excessive mitochondrial movement to produce a clustered mitochondrial distribution in the perinuclear region of cells (left). An excess of ARMC1 results in an aberrant enrichment of mitochondria at the cellular periphery (right).

The effect of DNAJC11 on ARMC1 membrane association may result from a faster off-rate of ARMC1 from DNAJC11 than from MIRO-MTFR complexes. An intriguing alternative possibility is that DNAJC11 actively facilitates ARMC1 removal from mitochondria by recruiting cytosolic Hsp70 via its N-terminal J domain to engage ARMC1. Potentially supporting this idea, Hsp70s are the primary cytosolic interactors of ARMC1 (fig. S16D) (*54*). In addition, purified HSPA8 (an Hsp70), but not a catalytically inactive D10N mutant (*55*), was sufficient to enhance ARMC1 release from cellular membranes in place of cytosol (fig. S16E). We speculate that the ability of DNAJC11 to promote ARMC1 release from the mitochondrial surface helps to balance the abundance and the assembly rate of ARMC1-MIRO-MTFR complexes, which is important for maintaining steady-state mitochondrial distributions (Fig. 7).

## DISCUSSION

We have elucidated the molecular interactions of ARMC1 that rationalize its role in maintaining steady-state mitochondrial distribution (Fig. 7). At the mitochondrial surface, ARMC1 facilitates the assembly of MIRO with MTFR and TMEM11, producing a previously unidentified complex that promotes mitochondrial localization toward the cellular periphery by specifically counteracting retrograde trafficking (Figs. 5 and 7). Hence, insufficient copy numbers of this complex result in collapsed mitochondrial distributions in the perinuclear region of cells, while too many copies result in the opposite extreme (Figs. 4, A and B, and 7). The critical abundance control of the ARMC1-MIRO-MTFR complex relies on the spatial partitioning of ARMC1, which is facilitated, in particular, through its association with a distinct mitochondrial complex containing DNAJC11 that promotes ARMC1 release from the mitochondrial surface (Figs. 6, E and F, and 7).

Our model helps to explain how changes in ARMC1 levels and partitioning influence mitochondrial distribution (Fig. 4, A and B). The cellular copy number of ARMC1 nearly matches the summed copy numbers of the MTFR and MIRO paralogs, and DNAJC11 is almost stoichiometric with ARMC1 (fig. S4A) (*20*, *21*). These closely matched stoichiometries suggest that small changes in ARMC1 expression and interactions can readily affect mitochondrial distribution. The propensity of ARMC1 to localize to the mitochondrial surface and to assemble into distinct protein complexes may additionally be modulated through posttranslational modifications on ARMC1 or its interactors (*26*, *56*).

Our findings also raise new questions. While we have outlined a model for how ARMC1 interactions affect the overall distribution of mitochondria in cells, ARMC1 and its interactors, particularly the MTFRs, also have heterogeneous impacts on mitochondrial morphology (*17*, *24*–*26*). Whether these factors directly regulate mitochondrial fission or fusion machinery or whether morphological defects result indirectly from aberrantly positioned mitochondria remains to be determined. In addition, detailed understanding of how DNAJC11 promotes ARMC1 removal from mitochondria and if similar functions apply more broadly to other organellar J-domain proteins await the development of strategies that are not affected by the pleiotropic effects and reduced cellular fitness observed upon prolonged DNAJC11 depletion (*46*, *57*, *58*). Last, how ARMC1 affects other putative functions linked to its interactors—such as TMEM11 (*27*), MTCH2 (*37*–*45*), or the MIB complex (*17*, *28*, *29*)—remains to be uncovered.

We have also identified functional and biochemical distinctions among individual MIRO, MTFR, and TRAK paralogs. We showed that TRAK2 depletion selectively reverses the ARMC1 KO phenotype in COS7 cells, while depletion of TRAK1 has no effect (Fig. 5, C to E). How this finding relates to how each TRAK paralog engages MIRO complexes or cytoskeletal motors is an important question for future investigation. In addition, while all three MTFR proteins interact with MIRO2, only MTFR1L stably binds MIRO1 and is destabilized by MIRO1 depletion (figs. S9, C and D, and S15C). This partnership may be especially relevant for mitochondrial trafficking through the long extensions of neurons, where these paralogs are specifically up-regulated (fig. S11B) (*49*). We also showed that MYO19 is not affected by ARMC1 but is specifically stabilized at the mitochondrial surface by MIRO2 rather than MIRO1 (fig. S15, A and C). It remains to be determined how these paralog-specific interactions and effects come into play in different cell types and during physiological processes such as mitosis or signaling cascades, where the expression levels and posttranslational modification status of different paralogs have been reported to differ (*9*, *26*, *50*, *53*).

Last, before our study, it was unknown how MTFRs localized to mitochondria or what factors they functioned with to affect mitochondrial morphology (*24*, *26*, *50*). Our findings establish ARMC1 as a dedicated MTFR biogenesis factor required for MTFR stability and complex assembly. Although MTFR has independent binding sites with both TMEM11 and MIRO, these interactions are not sufficient to maintain complex assembly or MTFR stability without ARMC1. Considering the diversity of mitochondrial surface proteins that lack generalizable targeting sequences, the role of ARMC1 in MTFR complex assembly may exemplify one of many bespoke protein biosynthetic mechanisms that operate at the surface of mitochondria. Together, by defining the specific interactions made by ARMC1 and selectively probing the impacts of each interaction on protein complex assembly and mitochondrial distribution, our work has uncovered molecular and conceptual insights into the mechanisms that actively regulate mitochondrial dynamics to maintain homeostasis.

## MATERIALS AND METHODS

### Plasmids, oligonucleotides, siRNAs, and antibodies

pX459 (Addgene, 62988) and guide RNA (gRNA)–AAVS1–T2 (Addgene, 41818), pOG44 (Invitrogen, V600520), pHDM-G (PlasmID, EvNO00061606), pHDM-HGPM2 (PlasmID, EvNO00061607), pHDM-tat1B (PlasmID, EvNO00061608), and pRC-CMV-rev1B (PlasmID, EvNO00061616) were obtained from the indicated suppliers. pDEST-hisMBP-AsCpf1-EC (Addgene, 79009) was modified to remove the maltose binding protein (MBP) sequence using standard molecular biology techniques. The insertion of target gRNAs into pX459 and the insertion of reporter sequences into pcDNA5/FRT/TO (Thermo Fisher Scientific) for incorporation into Flp-In T-REx cell lines, pHAGE for lentiviral generation, or an SP64-based vector for in vitro translation in a homemade rabbit reticulocyte lysate–based system (*22*) were all performed using standard molecular biology techniques such as Gibson assembly or restriction enzyme digestion and T4 ligation-based incorporation. Additional mutations were introduced using Phusion mutagenesis or Gibson assembly. All plasmids were validated by Sanger sequencing or whole-plasmid sequencing.

Details of oligonucleotides, small interfering (siRNAs), and antibodies are listed in Table 1.

**Table 1.** Key resources used in this study.

Oligonucleotide	Sequence
ARMC1 sgRNA	ACATCCCTGATCCTGGACGA
TMEM11 sgRNA	AGACCAGTTTGAGTACGAGC
MIRO1 sgRNA	AGCTCTATACTCACGTCCAC
MIRO2 sgRNA	CGCGACACCTTCGGGCAGGT
MTCH2 sgRNA	ACTCCCCAATATATACGTCT
ARMC1 sgRNA for H9 ES cells	TTTATGGACCATCCCAACCCTCCAGTCGC
5′ AAVS junction primers	CTCTAACGCTGCCGTCTCTC and TGGGCTTGTACTCGGTCATC
3′ AAVS junction primers	CACACAACATACGAGCCGGA and ACCCCGAAGAGTGAGTTTGC
siRNA	Catalog number
Control	Horizon D-001810-10-20
MTCH2	Horizon L-007371-00-0005
DNAJC11	Horizon L-021205-01-0005
MIRO1	Horizon L-010365-01-0005
MIRO2	Horizon L-008340-01-0005
TRAK1	Horizon L-020331-01-0005
TRAK2	Horizon L-014141-01-0005
TMEM11	Horizon L-005440-00-0005
MTFR1	Horizon L-019432-02-0005
MTFR1L	Horizon L-017152-00-0005
MTFR2	Horizon L-015495-02-0005
Antibody (usage)	Catalog number (identifier)
HSP60 [immunofluorescence (IF): 1:400]	Abcam ab128567 (RRID: AB_11145464)
HSP60 (IF: 1:400)	Abcam ab59457 (RRID: AB_2121285)
GOLGA2 (IF: 1:400)	Proteintech 11308-1-AP (RRID: AB_2115327)
TOM20 (IF: 1:400, WB: 1:5000)	Abcam ab186735 (RRID: AB_2889972)
FLAG (IF: 1:500)	Sigma-Aldrich F1804 (RRID: AB_262044)
FLAG–horseradish peroxidase (HRP) [Western blot (WB): 1:5000]	Sigma-Aldrich A8592 (RRID: AB_439702)
ARMC1 (WB: 1:2500)	Atlas HPA026085 (RRID: AB_1845031)
Calnexin (WB: 1:5000)	Enzo Life Sciences ADI-SPA-865 (RRID: AB_10618434)
MTFR1 (WB: 1:2500)	Proteintech 17778-1-AP (RRID: AB_2282301)
MTFR2 (WB: 1:1000)	Atlas HPA029792 (RRID: AB_10600210)
MTFR1L (WB: 1:1000)	Atlas HPA027124 (RRID: AB_10600211)
MAOA (WB: 1:1000)	Proteintech 10539-1-AP (RRID: AB_2137251)
FIS1 (WB: 1:5000)	Proteintech 10956-1-AP (RRID: AB_2102532)
OMP25 (WB: 1:1000)	Proteintech 15666-1-AP (RRID: AB_2201149)
MAVS (WB: 1:1000)	Cell Signaling Technology 3993 (RRID: AB_823565)
MIRO1 (WB: 1:1000)	Abcam ab55035 (RRID: AB_2269574)
MIRO1 (WB: 1:1000)	Abcam ab188029
MIRO2 (WB: 1:1000)	Abcam ab224089
MYO19 (WB 1:1000)	Proteintech 23906-1-AP (RRID: AB_2879357)
TRAK2 (WB 1:1000)	Proteintech 13770-1-AP (RRID: AB_2207961)
DNAJC11 (WB: 1:1000)	Proteintech 17331-1-AP (RRID: AB_2878385)
MTX1 (WB: 1:1000)	Cell Signaling Technology 29890
MTX2 (WB: 1:1000)	Atlas HPA031550 (RRID: AB_10599733)
MIC60 (WB: 1:5000)	Proteintech 10179-1-AP (RRID: AB_2127193)
HSPA8 (WB: 1:5000)	Proteintech 10654-1-AP (RRID: AB_2120153)
MTCH2 (WB: 1:2500)	Proteintech 16888-1-AP (RRID: AB_2266733)
MIC19 (WB: 1:1000)	Aviva Systems Biology ARP57040_P050 (RRID: AB_10639986)
MFN1 (WB 1:5000)	Proteintech 13798-1-AP (RRID: AB_2266318)
MFN2 (WB 1:5000)	Proteintech 12186-1-AP (RRID: AB_2266320)
OPA1 (1:2000)	BD Biosciences 612606 (RRID: AB_399888)
DRP1 (1:500)	Santa Cruz Biotechnology sc-271583 (RRID: AB_10659110)
SGTA (Small glutamine-rich tetratricopeptide repeat-containing protein alpha) (WB: 1:2500)	Gift from R. Hegde (*59*)
TOM70-HRP (WB: 1:500)	Santa Cruz Biotechnology sc-390545 (RRID: AB_2714192)
TIM23 (WB: 1:5000)	BD Biosciences 611222 (RRID: AB_398754)
TMEM11 (WB: 1:1000)	Proteintech 16564-1-AP (RRID: AB_2287468)
LRPPRC (WB: 1:5000)	Proteintech 21175-1-AP (RRID: AB_10733879)
BNIP3L (WB: 1:1000)	Atlas HPA015652 (RRID: AB_1845406)
Tubulin alpha (IF: 1:400)	Sigma-Aldrich T6199 (RRID: AB_477583)
Ubiquitin (WB: 1:100)	Santa Cruz Biotechnology sc-8017 (RRID: AB_628423)
Alexa Fluor 488–conjugated goat anti-mouse (IF: 1:500)	Jackson ImmunoResearch 115-545-003 (RRID: AB_2338840)
Alexa Fluor 594–conjugated goat anti-rabbit (IF: 1:500)	Jackson ImmunoResearch 111-585-003 (RRID: AB_2338059)
Alexa Fluor 647–conjugated alpaca anti-mouse (IF: 1:500)	Jackson ImmunoResearch 615-605-214 (RRID: AB_2721891)
Peroxidase-conjugated goat anti-mouse (WB: 1:5000)	Jackson ImmunoResearch 115-035-003 (RRID: AB_10015289)
Peroxidase-conjugated goat anti-rabbit (WB: 1:5000)	Jackson ImmunoResearch 111-035-003 (RRID: AB_2313567)

### Cell culture and editing

COS7 (American Type Culture Collection CRL-1651), Flp-In 293 T-REx (Invitrogen R78007), and Flp-In HeLa T-REx (gift from B. Raught) cells were maintained in Dulbecco’s modified Eagle’s medium (DMEM) with high glucose, GlutaMAX, and sodium pyruvate (Gibco, 10566016) supplemented with 10% fetal bovine serum (FBS) unless otherwise indicated at 37°C and 5% CO_2_. Expi293F cells were cultured in Expi293 medium (Gibco, A1435101) at 37°C and 8% CO_2_ with shaking at 120 rpm. No additional authentication was performed.

To generate KO cell lines, pX459 with target gRNA sequences identified using CHOPCHOP (*60*) was transfected using TransIT 293 (Mirus, 2706) for Flp-In 293 T-Rex cells or Lipofectamine 3000 (Invitrogen L3000) for Flp-In HeLa T-Rex and COS7 cells, according to the manufacturer’s instructions. Forty-eight hours after transfection, cells were placed under selection with puromycin (1 μg/ml; Gibco, A11138-03) for 48 hours. Clonal lines were isolated and validated with immunoblotting and genotyping. siRNA-mediated knockdowns were performed using reverse transfection by plating cells at 15 to 30% confluence into six-well plates containing 30 pmol of siRNA or 10-cm plates containing 120 pmol of siRNA with Lipofectamine RNAiMAX (Invitrogen, 13778-150) according to the manufacturer’s instructions 48 to 72 hours before analysis.

Reporters were integrated into the doxycycline-inducible Flp-In locus of Flp-In 293 T-REx and Flp-In HeLa T-REx cells by cotransfecting a 1:1 ratio of pOG44 and pcDNA5/FRT/TO containing the gene of interest using TransIT 293 (for Flp-In 293 T-Rex cells) or Lipofectamine 3000 (for Flp-In HeLa T-Rex cells) according to the manufacturer’s instructions. Cells were selected with blasticidin (2.5 μg/ml; Gibco, A11139-03) and hygromycin (50 μg/ml for Flp-In 293 T-Rex cells or 200 μg/ml for Flp-In HeLa T-Rex cells; MilliporeSigma, 31282-04-9) for 2 to 3 weeks, and the expression was validated by induction with doxycycline (100 ng/ml; Sigma-Aldrich, D9891) for 24 hours followed by immunoblotting. Constructs used to generate Flp-In 293 T-REx cells stably expressing 3xF-ARMC1 variants had the Promega-series CMV*d1* truncated promoter to obtain near-endogenous expression levels.

COS7 cells constitutively expressing 3xFlag-mNeonGreen3K-tagged versions of ARMC1 were generated using lentiviral transduction. To generate lentiviral particles, human embryonic kidney (HEK)–293T cells were transfected with a 2:1:1:1:10 ratio of pHDM-VSVG, pHDM-HGPM2, pHDM-tat1B, pRC-CMV-rev1B, and pHAGE containing the desired insert using TransIT 293 according to the manufacturer’s instructions. The medium was harvested 48 hours after transfection and used to transduce COS7 ARMC1 KO cells in the presence of polybrene (10 μg/ml; MilliporeSigma TR-1003-G). Forty-eight hours after transduction, cells were placed under selection with puromycin (1 μg/ml) for 48 hours. Cells were then sorted using a Sony SH800 Cell Sorter to isolate live, single cells only that were then sorted into low, medium, and high-expressing populations based on mNeonGreen3K signal excited using a laser at 488 nm and collected using a 525/50 emission filter. ARMC1 KO COS7 cells not expressing mNeonGreen3K were used to determine background fluorescence.

Human ES cells (H9, WiCell Institute) were cultured in E8 medium (*61*) on Geltrex-coated tissue culture plates with a daily medium change at 37°C with 5% CO_2_. Cells were passaged every 4 to 5 days with 0.5 mM EDTA in 1x Dulbecco’s phosphate-buffered saline (PBS). To generate ARMC1 KO H9 NGN2 ES cells, 80 pmol of Alt-R CRISPR-Cas12a crRNA (CRISPR RNA; IDT) was incubated with 63 pmol of AsCas12a protein (*62*) for 10 min at room temperature and electroporated into 2 × 10^5^ H9 NGN2 cells along with 39 pmol of Alt-R Cpf1 Electroporation Enhancer (IDT 1076301) using the Neon transfection system (Thermo Fisher Scientific). Mutants were identified by Illumina MiSeq and confirmed by immunoblotting.

For iNeuron differentiation, cells were plated at 2 × 10^4^/cm^2^ on Geltrex-coated plates in DMEM/F12 (Thermo Fisher Scientific, 11330057) supplemented with 1× N2 (Thermo Fisher Scientific, 17502048), 1x non-essential amino acids (NEAA; Life Technologies, 11140050), human brain-derived neurotrophic factor (BDNF; 10 ng/ml; PeproTech, 450-02), human Neurotrophin-3 (NT-3; 10 ng/ml; PeproTech, 450-03), mouse laminin (0.2 μg/ml; Cultrex; R&D Systems, 3446-005-01), 10 μM Y-27632 (PeproTech, 1293823), and doxycycline (2 μg/ml) on day 0. On day 1, Y-27632 was withdrawn. On day 2, the medium was replaced with Neurobasal medium (Thermo Fisher Scientific, 21103049) supplemented with 1x B27 (Thermo Fisher Scientific, 17504001) and 1x GlutaMAX (Thermo Fisher Scientific, 35050061) containing BDNF, NT-3, and doxycycline (1 μg/ml). Starting on day 4, half of the medium was replaced every other day. On day 7, cells were treated with Accutase (STEMCELL Technologies, 7920) and plated at 3 to 4 × 10^4^/cm^2^ on Matrigel-coated plates. Doxycycline was withdrawn on day 10.

### Recombinant protein purification

Purification of HSPA8 variants was carried out by transforming or BL21(DE3) *Escherichia coli* with a pGEX vector encoding an N-terminal GST-tag followed by a 3C protease cleavage site and the target sequence. Cells were grown in LB medium with ampicillin (100 μg/ml) to an *A*_600_ (absorbance at 600 nm) of 0.6 to 0.8 and then induced with 0.4 mM isopropyl-β-d-thiogalactopyranoside for 18 hours at 18°C. The cells were then collected and lysed in PBS supplemented with 1 mM dithiothreitol (DTT) and 1x protease inhibitor cocktail (PIC) by sonication. Clarified lysates after centrifugation at 34,541*g* for 20 min in a SS34 rotor (Sorvall) were passed through glutathione sepharose. The resin was washed once with PBS with 1 mM DTT followed by a wash with 50 mM tris (pH 8.0), 150 mM KCl, 10 mM MgCl_2_, 5 mM adenosine 5′-triphosphate (ATP), and 1 mM DTT and another with 50 mM tris (pH 8.0), 500 mM NaCl, and 1 mM DTT. Elutions were carried out with 50 mM tris (pH 8.8), 150 mM NaCl, and 25 mM reduced glutathione. Peak elutions were subjected to two rounds of dialysis into physiological salt buffer [PSB: 50 mM Hepes (pH 7.5), 100 mM KOAc, and 2.5 mM Mg(OAc)_2_] with 10% (v/v) glycerol and 1 mM DTT overnight at 4°C. GST-tagged 3C protease was added at 1:500 (v/v) after the first round of dialysis. GST-3C and cleaved GST were subtracted by passing the sample over glutathione sepharose. Purified proteins were aliquoted, frozen in liquid nitrogen, and stored at −80°C.

### Quantitative polymerase chain reaction

COS7 cells treated with siRNAs as described above were harvested by trypsinization and washed twice with cold PBS. Total RNA was isolated using an RNeasy kit (QIAGEN), according to the manufacturer’s instructions, followed by deoxyribonuclease I (QIAGEN) digestion, and RNA cleanup with the same RNeasy kit. First-strand cDNA was synthesized by reverse transcribing 200 to 500 ng of RNA with oligodT(20) primers and SuperScriptIII (Invitrogen), followed by an ribonuclease (RNase) H (Invitrogen) digest to remove leftover RNA. A reaction with no reverse transcriptase was included to control for genomic DNA contamination. Quantitative polymerase chain reaction (qPCR) was performed in a QuantStudio 7 Pro (Thermo Fischer Scientific) using PowerUp SYBR Green (Thermo Fisher Scientific) and primers annealing in different exons of the same transcript. A fourfold dilution standard curve was used to calculate amplification efficiencies of the different targets, and relative levels of transcripts were quantified using the ∆∆*C*t method (*63*). SRP14 was used as a reference gene control. Transcript ratios normalized to SRP14 were additionally normalized to the ratio in cells treated with control siRNAs. Three biological replicates, each with three to four technical replicates, were used for quantifications.

### Immunofluorescence

All images were collected with a Yokagawa CSU-X1 spinning-disk confocal microscope with Spectral Applied Research Aurora Borealis modification on a Nikon Ti2-E motorized inverted microscope equipped with a Plan Apo 100×/1.4 or a Plan Fluor 40×/0.4 numerical aperture (N.A.) oil immersion objective.

COS7 or Flp-In HeLa T-REx cells were grown in 24-well #1.5 coverslip-bottom plates (MatTek, P24G-1.5-13-F) to a confluence of 50 to 70%. Where indicated, cells were incubated with 100 nM MitoTracker CMXRos (Thermo Fisher Scientific, M7512) for 30 min before fixation. A total of 1 volume of 8% of formaldehyde (Electron Microscopy Sciences, 100503-917) in PBS was added to each well and incubated at 37°C for 30 min. After fixation, cells were washed with PBS, permeabilized with 0.1% Triton X-100 in PBS for 5 min, and then incubated with blocking solution 10% FBS in PBS with 0.05% Tween 20 (PBSt) for 30 min, followed by primary antibodies in blocking solution for either 1 hour at room temperature or at 4°C overnight. Cells were washed three times in PBSt, incubated with secondary antibodies in blocking solution for 1 hour in the dark, and washed three more times with PBSt. Samples were incubated with 4′,6-diamidino-2-phenylindole (DAPI; 1 μg/ml) solution (Thermo Fisher Scientific, D1306) at room temperature for 5 min in the dark and washed three more times with PBSt before imaging. Fluorescence was excited with solid-state lasers at 405 nm (for DAPI), 488 nm (for Alexa Fluor 488 or mNeonGreen3K), 561 nm (for Alexa Fluor 594 or MitoTracker CMXRos), and 640 nm (for Alexa Fluor 647) and collected using ET455/50m, ET525/50m or ET620/60m, and ET700/75m emission filters, respectively. Images were acquired with a Hamamatsu ORCA-Fusion-BT scientific complementary metal-oxide semiconductor (CMOS) camera and NIS Elements software. Z-series optical sections were collected with a step size of 0.5 μm (for Flp-In HeLa T-Rex) or 0.25 μm (for COS7), and single sections were selected from approximately the center of the cell.

To calculate distances between COMs, Z projections of each image were preprocessed by ImageJ/Fiji to separate different channels and to apply Otsu thresholding. Cells were manually segmented using the polygon selection tool, and the “clear outside” function was used to exclude signal from surrounding cells. Dividing cells and cells at the edges of fields of view were also excluded. The COM of each channel was then calculated using the scipy.ndimage.center_of_mass function from scypy v1.11.0. The distance between these centers of mass was calculated using a python function that gives the Euclidean distance between points *p* and *q* $\left[\sqrt{{\left(q_{1}-p_{1}\right)}^{2}+{\left(q_{2}-p_{2}\right)}^{2}}\right]$.

To assess the colocalization of F-ARMC1 with mitochondria, we first performed background subtraction of the images using the robust Otsu thresholding method (*64*). A region of interest was drawn around each cell in the Flag channel, and Manders’ colocalization coefficients (*65*) with mitochondria based on TOM20 staining were calculated using the ImageJ Coloc2 plugin with a point spread function of 2 and 25 randomizations.

To calculate the ratio of perinuclear to peripheral mitochondrial signal, Z projections of each image were split into constituent channels, normalized, and converted to an 8-bit format. For each field of view, binary nuclear masks were created using cv.THRESH_BINARY and cv2_THRESH_OTSU functions from OpenCv 3.4.2 and dilated using cv2.dilate using a 40 × 40 kernel. The ratio of the sum of the mitochondrial signal within the resulting perinuclear masks to the total mitochondrial signal in each field of view was then calculated. Plotting and statistical analysis of all microscopy data [one-way analysis of variance (ANOVA) and Tukey’s tests, unless otherwise indicated] were performed in GraphPad Prism v10.1.0.

### Live-cell confocal microscopy

COS7 or Flp-In 293 T-REx cells at 70% confluence were transfected with pcDNA5 FRT/TO vectors containing 3xFlag-tagged mNeonGreen3K appended to ARMC1 variants or ARMC1 CTD using Lipofectamine 3000 or TransIT 293 according to manufacturer’s instructions. Four hours after transfection, cells were trypsinized and replated onto six-well #1.5 MatTek coverslip-bottomed plates and grown for a further 24 hours. Cells were also incubated with 100 nM MitoTracker DeepRed (Thermo Fisher Scientific, M22426) for 30 min. Before analysis, cells were changed into FluoroBrite DMEM (Gibco, A1896701) containing 10% FBS. Cells were imaged as described above for immunofluorescence experiments but in an Okolab stage top incubation chamber set to 37°C with humidified 5% CO_2_. Fluorescence was excited with solid-state lasers at 405 nm (for TagBFP), 488 nm (for mNeonGreen3K), or 640 nm (for MitoTracker DeepRed) and collected using ET455/50m, ET525/50m, and ET700/75m emission filters, respectively.

Live-cell microscopy to analyze mitochondrial fission dynamics and of iNeurons was performed using a Yokogawa CSU-X1 spinning disk confocal on a Nikon Eclipse Ti-E motorized microscope. The system is equipped with a Tokai Hit stage top incubator, and imaging was performed at 37°C with 5% CO_2_ and 95% humidity equipped with 40×/0.4 N.A. Plan Apo Differential Interference Contrast (DIC) M N2 air and Nikon Plan Apo 60x/1.40 N.A. immersion oil objective lens. Fluorophores were excited in a sequential manner with a Nikon LUN-F XL solid state laser combiner (laser line–laser power): 405 nm–80 mW, 488 nm–80 mW, 561 nm–65 mW, and 640 nm–60 mW using a Semrock Di01-T405/488/568/647 dichroic mirror. Fluorescence emissions were collected through a Chroma ET455/50m (405 nm), Chroma ET525/36m (488 nm), Chroma ET 605/52m (561 nm), and a Chroma ET700/75m (for 640 nm) filters, respectively (Chroma Technologies). Images were acquired with a Hamamatsu ORCA-Fusion BT CMOS camera (6.5-μm^2^ photodiode, 16-bit) and NIS-Elements image acquisition software. Consistent laser intensity and exposure time were applied to all the samples, and brightness and contrast were adjusted equally by applying the same minimum and maximum display values in ImageJ/Fiji (*66*). All images in figures represent z-projections unless stated otherwise.

For quantitative analysis of mitochondrial fission/fusion dynamics, COS7 cells were seeded into μ-Slide eight-well glass bottom plates (ibidi 80807) and further cultured in the vessel until reaching appropriate confluency for microscopy. Cells were incubated for 1 hour at 37°C in DMEM containing Hoechst 33342 (Thermo Fisher Scientific, H1399), 250 nM MitoTrackerGreen (Thermo Fisher Scientific, M7514), and 1 μM SiR-tubulin (Spirochrome, SC002). Before microscopy, cells were washed in 1x PBS and imaged in FluoroBrite DMEM media. Cells were imaged with a Plan Apo 60×/1.40 N.A immersion oil objective lens with indicated laser power and exposure times (405: 30% 100 ms, 488: 20% 200 ms, 568: 20% 100 ms, 640: 100% 200 ms), and time courses (5 min) were recorded for later mitochondrial fission evaluation.

Neuronal precursor cells were seeded on Matrigel-coated FluoroDish cells (WPI FD35-100) during the differentiation protocol and maintained until the experimental time indicated in the figures. Cells were incubated for 1 hour at 37°C in ND2 containing Hoechst 33342, 250 nM MitoTrackerGreen or TMRE, and 1 μM SiR-tubulin. Before microscopy, cells were washed in 1x PBS, and medium was replaced. iNeurons were imaged over 5-min time lapses and 1-μm z-stacks using a Plan Apo 60×/1.40 N.A. immersion oil objective lens, with indicated laser power and exposure times (405: 30% 100 ms, 488: 20% 200 ms, 568: 20% 100 ms, 640: 100% 200 ms).

Axonal mitochondrial coverage was quantified in ImageJ/Fiji. The nuclear signal was squared, Gaussian-blurred (σ = 2), and converted into a mask using the “Huang” kernel, producing a dilated nuclear mask approximating the soma space of neurons. Mitochondrial objects, segmented based on MitoTrackerGreen, were subtracted from this soma mask, isolating axonal mitochondria. These axonal mitochondria were then quantified over the time course.

Mitochondrial membrane potential measurement was performed as follows: TMRE signal of t1 was used as a spatial mitochondrial mask for all mitochondrial objects in the timecourse. Filtered MitoTrackerGreen and TMRE channels were divided using the “imageCalculator” command in ImageJ/Fiji, and the resulting intensity ratios were measured throughout the series. The measurement of the first 10 time points was used for analysis, since later time points showed photobleaching in the TMRE channel. Measurements were saved as .csv files together with a .tiff image of the ratiometric image channel.

Mitochondrial fission rate quantification was performed in ImageJ/Fiji using composite, single-plane image series. The mitochondrial channel was duplicated for preprocessing, which included background subtraction and median filtering (radius = 2 px), followed by conversion into binary files (Huang threshold, black background). Mitochondrial objects were identified and quantified using the “Analyze Particles” function (size filter: 0.5 – infinity, summarize stack). An overlay of masks on the input image was generated and saved for visual verification purposes of the segmentation and analysis. Last, the summarized number of mitochondria per frame was saved as .csv files. The cumulative mitochondrial fission rate was then calculated as the sum of positive changes in mitochondrial object count across consecutive time points Σ(∆(*t*_*n*+1_ [#mitochondrial objects] − *t_n_*[#mitochondrial objects])).

### Isolation of cellular membranes and semi-permeabilized cells

Expi293F cells were grown in Expi293 media to 3 million cells/ml, or adherent cells were grown in DMEM to 80% confluence. Cells were harvested in cold PBS. Cells were centrifuged at 500*g* for 3 min at 4°C, resuspended in 10 pellet volumes of hypotonic buffer [20 mM Hepes (pH 7.4), 5 mM KCl, 1.5 mM MgCl_2_, 2 mM DTT, and 1x PIC], incubated on ice for 15 min, and lysed using 35 strokes in a tight-fitting glass-glass dounce homogenizer. Hepes (20 mM; pH 7.4), 525 mM mannitol, 175 mM sucrose, 5 mM EDTA, 2 mM DTT, and 1x PIC were then added. The lysate was centrifuged twice at 700*g* for 10 min to remove nuclei and unbroken cells. The supernatant was then centrifuged at 8500*g* in an SS34 rotor (Sorvall) for 10 min to pellet organelles. This membrane fraction was resuspended in MSH buffer [20 mM Hepes (pH 7.4), 210 mM mannitol, 70 mM sucrose, 0.5 mM EDTA, 2 mM DTT, and 1x PIC] and either aliquoted and snap-frozen or used immediately for F-ARMC1 binding or UV crosslinking assays. For protease protection assays, membranes (at a protein concentration of approximately 5 mg/ml) were incubated on ice for 1 hour without or with proteinase K (PK; 0.5 mg/ml) or PK (0.5 mg/ml) plus 0.1% Triton X-100. PK activity was inhibited with the addition of 5 mM phenylmethylsulfonyl fluoride on ice for 5 min, followed by 5 volumes of boiling SDS–polyacrylamide gel electrophoresis (SDS-PAGE) sample buffer.

To prepare semi-permeabilized cells, Flp-In 293 T-REx cells were grown to 80% confluence in a 10-cm plate. Medium was aspirated, and the cells were carefully washed in cold PBS on the plate. A 1 ml of cold 0.02% digitonin (MilliporeSigma, 11024-24-1) in RNC buffer [50 mM Hepes (pH 7.4), 100 mM KOAc, and 5 mM Mg(OAc)_2_] was layered onto the cells and incubated at 4°C for 10 min. The semi-permeabilized cells were harvested in 10 ml of RNC buffer and pelleted at 1000*g* for 3 min at 4°C. The pelleted semi-permeabilized cells were washed again with RNC buffer, pelleted at 13,000*g* for 30 s at 4°C, resuspended in a volume of RNC buffer equivalent to a concentration of 100,000 cells/μl, and used immediately for crosslinking or release assays.

### Cellular fractionations, F-ARMC1 purifications, and sucrose gradients

Cells were incubated with 0.02% digitonin in RNC buffer containing 250 mM sucrose and 1x PIC at a ratio of approximately 50 μl of buffer per million cells, incubated on ice for 10 min, and centrifuged at 1000*g* for 5 min at 4°C. The supernatant (cytosolic fractions) was collected, and the pellet (organellar membranes) was washed once in RNC buffer containing 250 mM sucrose. For immunoblotting, the washed pellet was solubilized with 1% digitonin in RNC buffer with 1x PIC at a volume equivalent to the semi-permeabilization step, incubated on ice for 10 min, and centrifuged at 21,000*g* for 5 min at 4°C before analyzing the solubilized material. Because enzyme-catalyzed chemiluminescent signals are not linear, we do not quantify immunoblotting outputs. Immunoblots of fractionations were controlled for by running samples that are directly compared on the same blots and probing for fractionation controls, such as the cytosolic protein SGTA and the mitochondrial protein TOM20. All fractionation experiments shown are representative of at least three independent replicates.

For large-scale affinity purifications of F-ARMC1 to identify interactors, 48 15-cm plates of Flp-In 293 T-Rex F-ARMC1 rescue cells were grown to 90% confluence in DMEM containing doxycycline (100 ng/ml) for 48 hours. Membrane-associated F-ARMC1 was separated from cytosolic F-ARMC1 as described above. The resulting membrane pellet was resuspended in 6 volumes of RNC buffer containing 1% digitonin and 1x PIC, incubated on ice for 10 min, and centrifuged at 21,000*g* for 5 min. M2 anti-Flag affinity resin (Sigma-Aldrich, A2220) was added to the supernatant and incubated at 4°C with constant rotation for 1.5 hours. The resin was washed 4× in RNC buffer with 0.25% digitonin and subjected to sequential elutions by incubation with 3xFlag peptide (0.5 mg/ml; APExBIO MSPP-A6001) in RNC buffer with 0.25% digitonin. Peak fractions were analyzed directly or pooled and concentrated using a 10,000 MWCO cassette before analysis by SDS-PAGE and Coomassie staining or immunoblotting.

For small-scale copurification analyses, membranes from cells expressing different F-ARMC1 variants were prepared as above, solubilized in 1% digitonin in RNC buffer with 1x PIC on ice for 10 min, and centrifuged at 21,000*g* for 5 min at 4°C. Solubilized material was diluted to 1 ml with 0.1% digitonin in RNC buffer and incubated with 10 μl of M2 resin for 1 hour at 4°C with constant rotation. The resin was washed four times in with 0.1% digitonin in RNC buffer, and the bound proteins were eluted with SDS-PAGE sample buffer.

To analyze the approximate native sizes of complexes involving ARMC1, 20 μl of solubilized membranes isolated from cells expressing F-ARMC1 or F-ARMC1 affinity purifications as described above were applied to a 10 to 30% sucrose gradient consisting of five equal steps of 40 μl of 30, 25, 20, 15, and 10% sucrose in PSB containing 0.25% digitonin. The gradients were centrifuged at 55,000 rpm for 1 hour at 4°C in a TLS-55 rotor (Beckman Coulter), followed by manual collection of 11 20-μl fractions.

### In vitro ARMC1 binding assays

In vitro transcription reactions of F-ARMC1 variants were performed with SP6 polymerase in a homemade transcription mix (*22*) for 1 hour at 37°C and used directly for in vitro translation in a homemade rabbit reticulocyte-based system (*22*) at 32°C in the absence of organelles for 20 min before the addition of RNase A (50 μg/ml; Sigma-Aldrich, 10109142001) to stop translation. For experiments as in Fig. 1D, ^1^/_10_ of the translation reaction volume of organelles or MSH buffer was added, and the samples were incubated at 37°C for another 20 min. Membrane-bound material was isolated by layering the reaction onto 15 reaction volumes of 20% sucrose in PSB and centrifuging at 21,000*g* for 10 min in a benchtop centrifuge at 4°C. The pelleted material was resuspended in 1% SDS in 0.1 M tris (pH 8.0), added to SDS-PAGE sample buffer, and analyzed directly by SDS-PAGE and autoradiography. The quantification of autoradiography from film scans was performed using ImageJ. After background subtraction, the ratio of radiolabeled F-ARMC1 signal in the pellet fraction to the total reaction was calculated and normalized to the values obtained with WT F-ARMC1. The normalized ratios from independent experiments were averaged and reported with the SEM and individual values in all plots. Plotting of autoradiography quantifications and statistical analysis (one-way ANOVA and Tukey’s test) was performed using GraphPad Prism v10.1.0.

### Fluorescent flow cytometry

Cells expressing MTFR2-mTagBFP-P2A-mCherry3V and treated with 1 μM inhibitors indicated for 4 hours at 37°C were detached using 0.25% trypsin-EDTA, collected in complete growth medium, and centrifuged at 500*g* for 3 min at 4°C. The pelleted cells were washed and collected in PBS containing 1% FBS and filtered through a 35 mm mesh strainer. Data were collected on an Attune NxT flow cytometer. Samples were gated to analyze live, single cells only. Fluorescence was excited with lasers at 405 nm (for mTagBFP) and 561 nm (for mCherry3V) and collected using 440/50 and 615/20 emission filters, respectively. Data analysis was performed using FlowJo.

### Crosslinking assays

For photocrosslinking experiments, transcripts of F-ARMC1 variants containing the UAG stop codon at the desired position of Bpa incorporation were generated using a homemade transcription system and SP6 polymerase and used to program in vitro translation reactions in a homemade rabbit reticulocyte lysate–based system (*22*) supplemented with an amber stop codon suppression system (*36*), as described above, at 32°C for 20 min before the addition of RNase A (50 μg/ml). One-tenth of the translation reaction volume of organelles or one-fifth of the translation reaction volume of semi-permeabilized cells were added, and the samples were incubated at 37°C for 20 min. For samples containing organelles, the organellar membranes were isolated by layering the reaction onto 20% sucrose in PSB, centrifuging at 21,000*g* for 10 min in a benchtop centrifuge at 4°C, and resuspending the pellet in the reaction of volume of PSB with 1 mM DTT and 250 mM sucrose. Reactions containing semi-permeabilized cells were diluted 10-fold in PSB with 1 mM DTT and 250 mM sucrose. All samples were crosslinked on a chilled metal block ~10 cm under a UVP B-100 series lamp for 10 min. For immunoprecipitations, SDS was added to the reaction to a final concentration of 1%, and the samples were heated to 95°C for 3 min and then cooled down to room temperature. The supernatant was diluted 10-fold in immunoprecipitation (IP) buffer, and 10-μl packed volume of either M2 anti-Flag affinity resin or protein A resin plus approximately 2 μg of each target antibody was added and mixed at 4°C for 1 hour. The resin was washed three times with IP buffer, and the samples were eluted by the addition of sample buffer and analyzed by SDS-PAGE and autoradiography.

For chemical crosslinking, F-ARMC1 variants were transcribed, translated, and incubated with semi-permeabilized cells or organellar membranes as described above, except without the presence of the suppression mix during the translation reaction. The samples were then diluted 10-fold in RNC buffer, and 250 μM BMH (Thermo Fisher Scientific, 22330) or the equivalent volume of DMSO (Sigma-Aldrich, 472301) was added. The reactions were incubated on ice for 1 hour. For the analysis of total crosslinks, the samples were quenched directly in SDS-PAGE sample buffer. For immunoprecipitations, the samples were quenched with 2.5 mM DTT and 1% SDS and heated at 95°C for 5 min. The samples were then diluted 10-fold in PBS with 0.5% Triton X-100 and rotated overnight with 2 μg of the target antibody and 10-μl packed volume of protein A resin (Repligen, CA-PRI-1000). The resin was washed three times in PBS with 0.5% Triton X-100, and the samples were eluted by the addition of SDS-PAGE sample buffer and analyzed by SDS-PAGE and phosphor imaging using a Typhoon imager (Cytiva). Because phosphor imaging does not readily allow the direct alignment of stained molecular weight markers on the dried gel with the radioactive signal, molecular weight markers for these panels are not indicated.

### ARMC1 membrane release assays

To isolate cytosol for supplementation in release assays, Flp-In 293 T-REx cells were grown to 80% confluence in three 15-cm plates; washed once with cold PBS; centrifuged at 500*g* for 3 min at 4°C; resuspended in 800 μl of 10 mM Hepes (pH 7.5), 10 mM KOAc, 1.5 mM Mg(OAc)_2_, 1 mM DTT, and 1x PIC; and swelled on ice for 30 min. Cells were lysed by passage through a 26-gauge syringe needle eight times, and KOAc was added to the lysate to a concentration of approximately 100 mM. The lysate was centrifuged at 100,000 rpm in a TLA120.2 rotor (Beckman Coulter) for 40 min at 4°C, and the supernatant (cytosol, at approximately 5 mg/ml) was aliquoted and snap-frozen before use.

The membrane fractions from Flp-In 293 T-REx cells (treated with either control siRNA or siRNA against DNAJC11) or WT or 4K F-ARMC1 rescue cells were prepared as described above. The membranes from the equivalent of 350,000 cells were added to 15-μl reactions containing 10 μl of RNC buffer, cytosol isolated from Flp-In 293 T-REx cells as described above or 15 μM recombinant WT or D10N HSPA8 (Hsc70), and 1 mM ATP. The reactions were incubated at 32°C for 15 min before centrifugation at 1000*g* for 5 min at 4°C. The supernatant containing material released from the membranes was collected, and the pellet washed once in RNC buffer containing 250 mM sucrose before being solubilized in SDS-PAGE sample buffer.

For release assays of radiolabeled F-ARMC1, in vitro translations were performed in the absence of membranes as described for the ARMC1 binding assays and stopped with the addition of RNase A (50 μg/ml). One-fifth of the translation reaction volume of semi-permeabilized cells (from Flp-In 293 T-REx cells treated with either control siRNA or siRNA against DNAJC11) was added, and the samples were incubated at 37°C for 20 min. The semi-permeabilized cells were re-isolated by spinning at 1000*g* for 5 min at 4°C, washed, and then resuspended in the equivalent starting volume of RNC buffer containing 250 mM sucrose. The radiolabeled F-ARMC1-loaded membranes were then used for release assays as described above. Quantification was performed from phosphor imaging using ImageQuant TL analysis software. After background subtraction, the ratios of F-ARMC1 signal in the respective supernatant and pellet fractions were directly calculated.

### Analysis of Alphafold models

Alphafold 2.2.0 multimer (*67*) predictions were run on the O2 cluster supported by Research Computing at Harvard Medical School using a max template date of 01-01-2022 and one or two predictions per model. Alphafold3 predictions were run using the web-based server (*47*). All interactions discussed and analyzed in this study were predicted with both Alphafold 2.2.0-multimer and Alphafold3, except for the putative interaction between the ARMC1 C terminus and TMEM11, which was only observed with Alphafold3 predictions. All visualization, analysis, and generation of figure panels of the models and predicted aligned error (PAE) plots were done in ChimeraX (*68*) v1.5. For the interactions discussed in the text, all five models were examined, and the highest-ranking models (based on pLDDT values) were used for figure generation.

### Multiplexed mass spectrometry

For whole-cell proteomic analyses (tables S1 and S2), cells were washed twice with 1x PBS, harvested on ice using a cell scraper in 1x PBS, pelleted via centrifugation for 5 min at 5000*g* at 4°C, and washed with 1x PBS. For analyses of membrane fractions from MIRO KO and rescue cells (tables S4 and S5), organellar membranes were obtained by incubating cells with 0.02% digitonin in RNC buffer containing 250 mM sucrose and 1x PIC at a ratio of approximately 50 μl of buffer per million cells, incubated on ice for 10 min, and centrifuged at 1000*g* for 5 min at 4°C. The supernatant (cytosolic fractions) was collected, and the pellet (organellar membranes) was washed once in RNC buffer containing 250 mM sucrose.

Sample preparation for proteomic analysis was performed according to previously published studies (*49*, *69*). Cells and membranes were resuspended in 8 M urea, 150 mM tris (pH 7.4), and 150 mM NaCl with protease and phosphatase inhibitors (Roche, 4906845001). After 10 s of sonication and optional passage through a 25-gauge needle, lysed cells were pelleted, and the protein concentration of clarified sample was determined using a bicinchoninic acid (BCA) kit (Thermo Fisher Scientific, 23225). A 100 μg of each sample was incubated for 30 min at 37°C with 5 mM tris(2-carboxyethyl) phosphine (TCEP; Gold Biotechnology) for disulfide bond reduction with subsequent alkylation with 25 mM chloroacetamide (Sigma-Aldrich, C0267) for 10 min at room temperature with gentle shaking. MeOH-chloroform precipitation of samples was performed as follows: to each sample, MeOH (four parts) was added followed by vortexing, chloroform (one part) was added followed by vortexing, and last H_2_O (three parts) was added. After vortexing, the suspension was centrifugated for 2 min at 14,000*g*, and the aqueous phase around the protein precipitate was removed using a loading tip. The precipitate was washed twice with MeOH and resuspended in 200 mM 3-[4-(2-Hydroxyethyl)piperazin-1-yl]propane-1-sulfonic acid (EPPS, pH 8.0; Sigma-Aldrich, E9502) and digested for 2 hours with LysC (1:100) at 37°C, followed by Trypsin (Promega, V511C) digestion (1:100) at 37°C overnight with gentle shaking.

Digested samples (50 μl) were labeled by adding 10 μl of TMT reagent (Thermo Fisher Scientific, A52045, stock: 20 mg/ml in acetonitrile (ACN); MilliporeSigma, 34851] together with 10 μl of ACN to yield a final ACN concentration of approximately 30% (v/v) for 2 hours at room temperature before quenching the reaction with hydroxylamine (Thermo Fisher Scientific, 90115) to a final concentration of 0.5% (v/v) for 15 min. The TMTpro-labeled samples were pooled together at a 1:1 ratio, resulting in consistent peptide amount across all channels. Pooled samples were vacuum-centrifuged for 1 hour at room temperature to remove ACN, followed by reconstitution in 1% formic acid (FA; Sigma-Aldrich, 94318), desalting using C18 solid-phase extraction (200 mg; Sep-Pak, Waters, WAT054960), and vacuum centrifugation until near dryness. Dried peptides were resuspended in 10 mM NH_4_HCO_3_ (pH 8.0) and fractionated using basic pH reverse phase HPLC. Samples were offline fractionated into 96 fractions over a 90-min run by using an Agilent LC1260 with an Agilent 300 Extend C18 column (3.5-μm particles, 2.1-mm inner diameter, and 250 mm in length) with mobile phase A containing 5% ACN and 10 mM NH_4_HCO_3_ in liquid chromatography–MS (LC-MS) grade H_2_O, and mobile phase B containing 90% ACN and 10 mM NH_4_HCO_3_ in LC-MS grade H_2_O (both pH 8.0). The 96 resulting fractions were then pooled in a noncontinuous manner into 24 fractions (*70*). This set of 24 fractions was divided into 2 × 12 sets, acidified by addition of 1% FA, and vacuum-centrifuged until near dryness. One set (12 samples) was desalted via StageTip, dried, and reconstituted in 10 μl of 5% ACN and 5% FA before LC-MS/MS processing.

Proteomics data collection for Flp-In 293 T-REx cells and membrane fractions were performed on a Orbitrap Eclipse or Orbitrap Exactive HF-X hybrid mass spectrometer (Thermo Fisher Scientific), coupled with a field asymmetric ion mobility spectrometry (FAIMS) Pro device and a Proxeon EASY-nLC1200 liquid chromatography (Thermo Fisher Scientific). A 10% of resuspended samples was loaded on a 35-cm analytical column (100-mm inner diameter) packed in-house with Accucore150 resin (150 Å, 2.6 mm; Thermo Fisher Scientific) for LC-MS analysis. Peptide separation was performed with a gradient of ACN (0.1% FA) from 3 to 13% (0 to 83 min) and 13 to 28% (83 to 90 min) during a 90-min run. LC-MS/MS was combined with three optimized compensation voltages (CVs) parameters on the FAIMS Pro Interface to reduce precursor ion interference (*71*). Data-dependent acquisition (DDA) was performed by selecting the most abundant precursors from the MS1 stage for each CV (−40/−60/−80 V) for MS/MS analysis over a 1.25-s duty cycle. The parameters for MS1 scans in the Orbitrap include a 400 to 1600 mass/charge ratio (*m*/*z*) mass range at 60,000 resolution [at 200 Thomson (Th)] with 4 × 10^5^ automated gain control (AGC) (100%), and a maximum injection time (max IT) of 50 ms. Most abundant precursors [with 120-s dynamic exclusion ± 10 parts per million (ppm)] were selected from MS1 scans, isolated using the quadrupole (0.6-Th isolation), fragmented with higher-energy collisional dissociation (36% collision energy), and subjected to MS/MS (MS2) in the Orbitrap detector at 50,000 resolution, 5 × 10^5^ AGC, 110 to 200-Th first mass, and a maximum ion time of 86 ms.

For whole-cell proteomics of Flp-In HeLa T-REx cells, the same FAIMS and MS1 parameters were implemented (with 1.25-s duty cycle/CV for DDA) with the Multi-Notch synchronous-precursor-selection (SPS)–MS3 acquisition method (*72*) to further reduce ion interference in TMT reporter quantification (*70*). Most abundant precursors (with 120-s dynamic exclusion ± 10 ppm) were selected from MS1 scans, isolated using the quadrupole (0.6 Th isolation), fragmented with collision-induced dissociation at 35 normalized collision energy (NCE), and subjected to MS/MS in the ion trap (turbo scan speed, 35-ms max IT, and 1.0 × 10^4^ AGC). Using Real Time Search analysis software (*73*, *74*), an SPS API-MS3 scan was collected on the top 10 most intense b- or y-ions from the matched peptide identification (determined by an online search of its respective MS/MS scan). MS3 scans were performed on the Orbitrap (AGC of 2.0 × 10^5^, NCE of 55, max IT of 250 ms, and 50,000 resolution at 200 Th). To increase quantitative sampling (MS3 scans) of proteins during each MS injection, a two peptide per protein per sample closeout was set. This ensures no more than two peptide-spectrum matches per protein (that pass quality filters) are subjected to MS3 scans, reducing redundant protein MS sampling and potentially increasing proteome depth (*74*).

Raw mass spectra were converted to mzXML, monoisotopic peaks reassigned using Monocle (*75*), and searched using Comet (*76*) against all canonical isoforms found in the Human reference proteome database (UniProt Swiss-Prot, 2019-01) as well as against sequences from commonly found contaminant proteins and reverse sequences of proteins as decoys for target-decoy competition (*77*). For searches, a 50-ppm precursor ion tolerance and 0.9-Da product ion tolerance for ion trap MS/MS as well as trypsin endopeptidase specificity on C-terminal with two maximum missed cleavages were set. Static modifications were set for carbamidomethylation of cysteine residues (+57.021 Da) and TMTpro labels on lysine residues and N termini of peptides (+304.207 Da); variable modification was set for oxidization of methionine residues (+15.995 Da). Peptide-spectrum matches were filtered at 2% false discovery rate (FDR) using linear discriminant analysis (Picked FDR method, based on XCorr, DeltaCn, missed cleavages, peptide length, precursor mass accuracy, fraction of matched product ions, charge state, and number of modifications per peptide) (additionally restricting PSM Xcorr >1 and peptide length > 6 (*78*), and after a 2% protein FDR target filtering (*79*), PSM reporter ion intensities were quantified. Quantification was performed using a 0.003-Da window around the theoretical TMT-reporter *m*/*z* and filtered on precursor isolation specificity of >0.5 in the MS1 isolation window and for CORE output filtered by summed signal-to-noise (SNR) across all TMT channels >100.

MSstatsTMT (*80*) was performed on peptides with >200 summed SNR across TMT channels. For each protein, the filtered peptide-spectrum match TMTpro raw intensities were summed and log_2_-normalized to calculate protein quantification values (weighted average) and normalized to total TMT channel intensity across all quantified PSMs (adjusted to median total TMT intensity for the TMT channels) (*81*). Log_2_-normalized summed protein reporter intensities were compared using a Student’s *t* test, and *P* values were corrected for multiple hypotheses using the Benjamini-Hochberg adjustment (*82*).
