# Supplementary material for: ARMC1 partitions between distinct complexes and assembles MIRO with MTFR to control mitochondrial distribution
Source: Sci Adv. 2025 Apr 9;11(15):eadu5091. doi: 10.1126/sciadv.adu5091 (PMC11980836; doi:10.1126/sciadv.adu5091)
Supplement: Supplementary file 1 — Figs. S1 to S16 Legends for tables S1 to S5 Legend for movie S1 [file sciadv.adu5091_sm.pdf]

Science Advances
AAAS

Supplementary Materials for

ARMC1 partitions between distinct complexes and assembles MIRO with MTFR to control mitochondrial distribution

Michael J. McKenna *et al.*

Corresponding author: Sichen Shao, sichen_shao@hms.harvard.edu

Sci. Adv. **11**, eadu5091 (2025)
DOI: 10.1126/sciadv.adu5091

The PDF file includes:

Figs. S1 to S16
Legends for tables S1 to S5
Legend for movie S1

Other Supplementary Material for this manuscript includes the following:

Tables S1 to S5
Movie S1

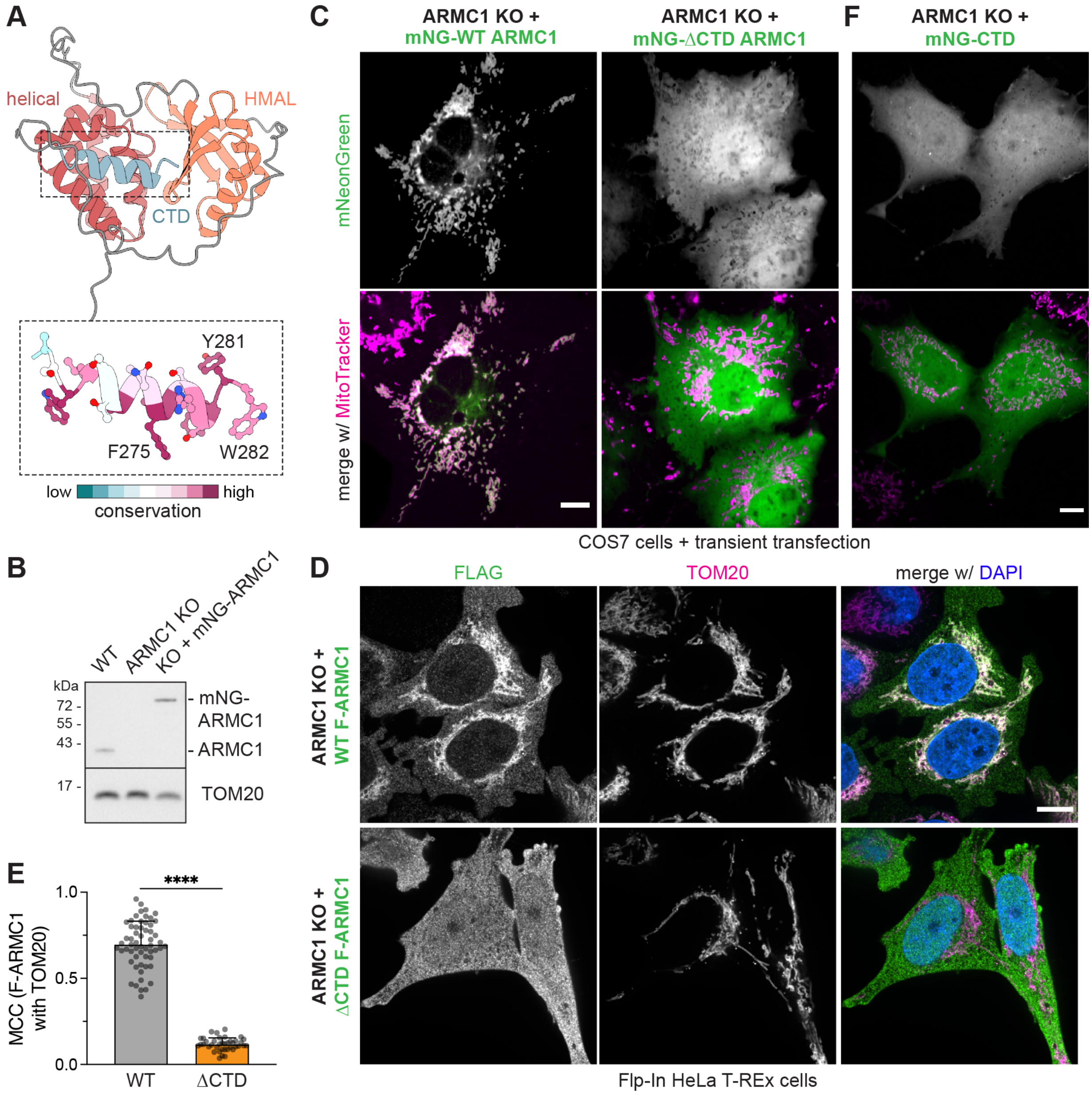

Fig. S1. The ARMC1 CTD mediates mitochondrial localization.

(A) Alphafold model of ARMC1 (top) colored according to domain: helical (dark red), heavy-metal-associated-like (HMAL; salmon), and C-terminal domain (CTD, light blue). The inset shows the C-terminal amino acids colored by conservation.

(B) SDS-PAGE and immunoblotting of wildtype (WT) and ARMC1 knockout (KO) COS7 cells without or with stable re-expression of near-endogenous levels of mNeonGreen (mNG)-tagged ARMC1 introduced by lentiviral transduction and then subjected to fluorescence activated cell sorting (FACS) based on mNG fluorescence levels.

(C) The ARMC1 CTD is necessary for mitochondrial localization. Live-cell images of ARMC1 KO COS7 cells transfected with WT or ΔCTD mNG-ARMC1 (green) and stained with MitoTracker (magenta). Scale bar, 10 μm.

(D) Immunofluorescence of ARMC1 KO Flp-In HeLa T-REx cells re-expressing WT or ΔCTD Flag-tagged ARMC1 (F-ARMC1), showing the Flag signal of F-ARMC1 (green), the mitochondrial protein TOM20 (magenta), and nuclei (DAPI, blue). Scale bar, 10 µm.

(E) Manders' colocalization coefficients (MCC, mean + s.d. and measurements for n=55 WT and n=39 ΔCTD cells) of the F-ARMC1 variants with TOM20 as in (D) show that the ARMC1 CTD is required for mitochondrial localization. ****, $p<0.0001$.

(F) The ARMC1 CTD is not sufficient for mitochondrial localization. As in (C) with transfected mNG-CTD (mNeonGreen appended to the ARMC1 CTD). Scale bar, 10 µm.

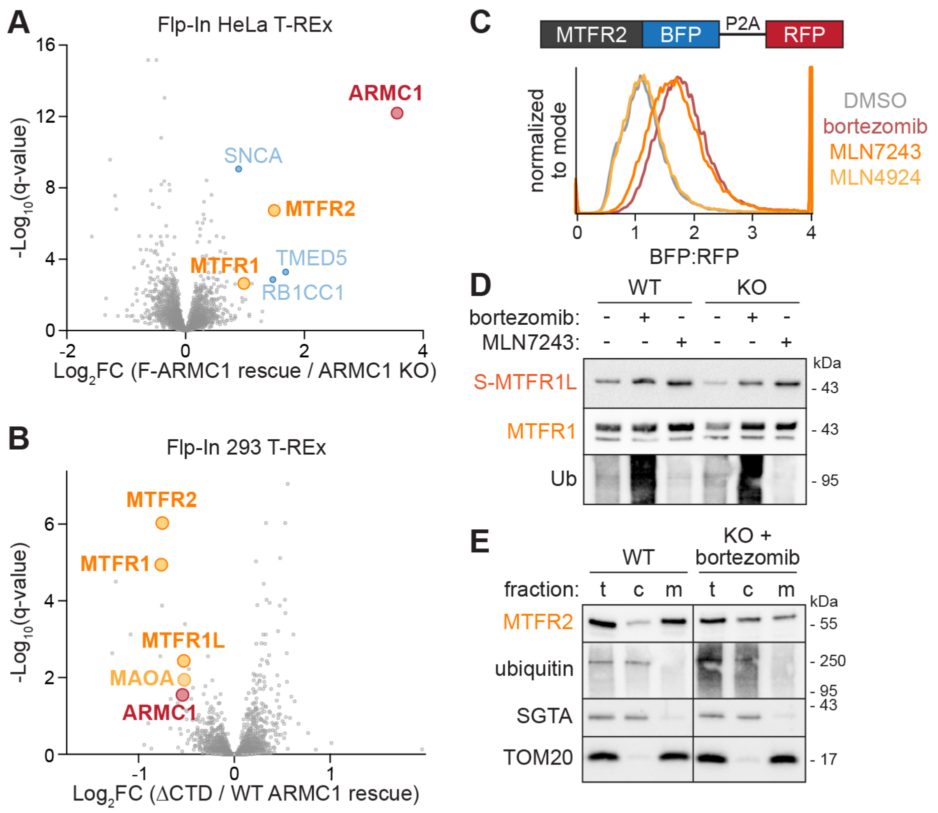

Fig. S2. ARMC1 prevents MTFR degradation.

(A) MTFR levels depend on ARMC1. Volcano plot of multiplexed proteomics data showing fold-change (FC) of protein levels in ARMC1 knockout (KO) Flp-In HeLa T-REx cells without or with rescue with Flag-tagged ARMC1 (F-ARMC1).

(B) Volcano plot showing protein level changes in ARMC1 KO Flp-In 293 T-REx cells re-expressing ΔCTD versus wildtype (WT) F-ARMC1.

(C) Inhibitors of the ubiquitin-proteasome system stabilize MTFR. Flp-In 293 T-REx cells expressing a BFP-tagged MTFR2 reporter separated from RFP synthesized from the same transcript through a P2A ribosome skipping site were treated with the 1 μM of the indicated inhibitor (bortezomib, a proteasome inhibitor, MLN7243, a ubiquitin-activating enzyme inhibitor, or MLN4924, a NEDD8-activating enzyme inhibitor) for 4 hours and analyzed by fluorescent flow cytometry.

(D) Immunoblotting of lysates from WT or ARMC1 KO Flp-In 293 T-REx cells expressing Strep-tagged MTFR1L (S-MTFR1L) treated with 1 μM bortezomib or MLN7243 for 4 hours. Note: stabilization of S-MTFR1L and endogenous MTFR1 in ARMC1 KO cells with the ubiquitin-proteasome system inhibitors.

(E) Immunoblotting of cellular fractionations of untreated WT Flp-In 293 T-REx cells or ARMC1 KO cells treated with 1 μM bortezomib for 4 hours shows increased MTFR2 in the cytosolic fraction of bortezomib-treated ARMC1 KO cells. t, total; c, cytosolic fraction, m, membrane fraction.

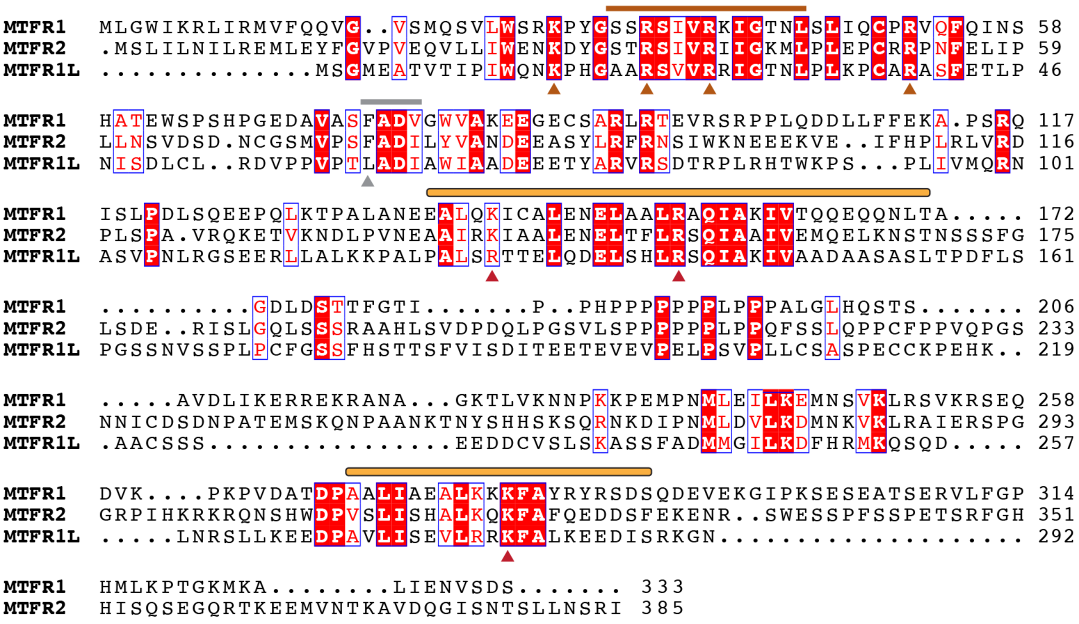

Fig. S3. MTFR sequence alignments.

Sequence alignment of human MTFR1, MTFR2, and MTFR1L. The residues (brown arrows) and regions (brown line) involved in TMEM11 interaction, the helices at the ARMC1 interaction interface (light orange rods), key residues involved in ARMC1 interaction (red arrows), and the region implicated in MIRO interaction (gray arrow and line) are indicated.

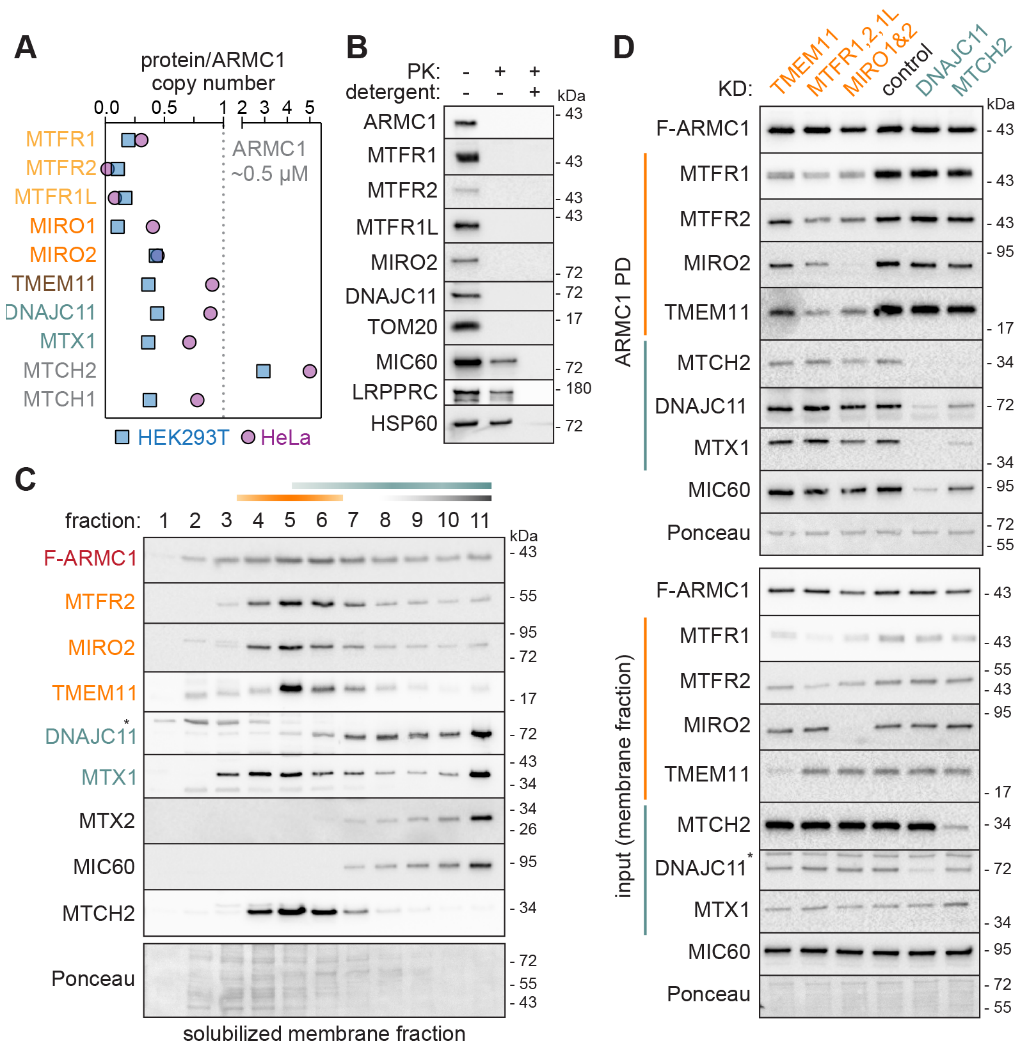

Fig. S4. ARMC1 interacts with distinct mitochondrial complexes.

(A) Relative abundance of the indicated proteins compared to ARMC1 (gray dotted line, estimated to be ~0.5 µM) based on mass spectrometry analyses of HEK293T (ref. *20*) and HeLa (ref. *21*) cell lysates.

(B) Cellular membranes treated without or with 0.5 mg/mL proteinase K (PK) in the absence or presence of 0.1% Triton X-100 (detergent) were analyzed by SDS-PAGE and immunoblotting. Note: PK digests peripheral and outer mitochondrial membrane (OMM) proteins, including ARMC1, MTFRs, MIRO, and DNAJC11. Proteins that reside in the intermembrane space, inner mitochondrial membrane, and mitochondrial matrix are protected from PK digestion by the OMM unless it is solubilized by detergent.

(C) Immunoblotting of size fractionations of detergent-solubilized organelles from F-ARMC1 Flp-In 293 T-REx rescue cells over a 10-30% sucrose gradient.

(D) ARMC1 partitions into independent mitochondrial protein complexes. Immunoblotting of membrane fractions (input, bottom) and F-ARMC1 pulldowns (PD, top) from rescue Flp-In 293 T-REx cells treated with siRNAs to knock down (KD) the indicated component(s).

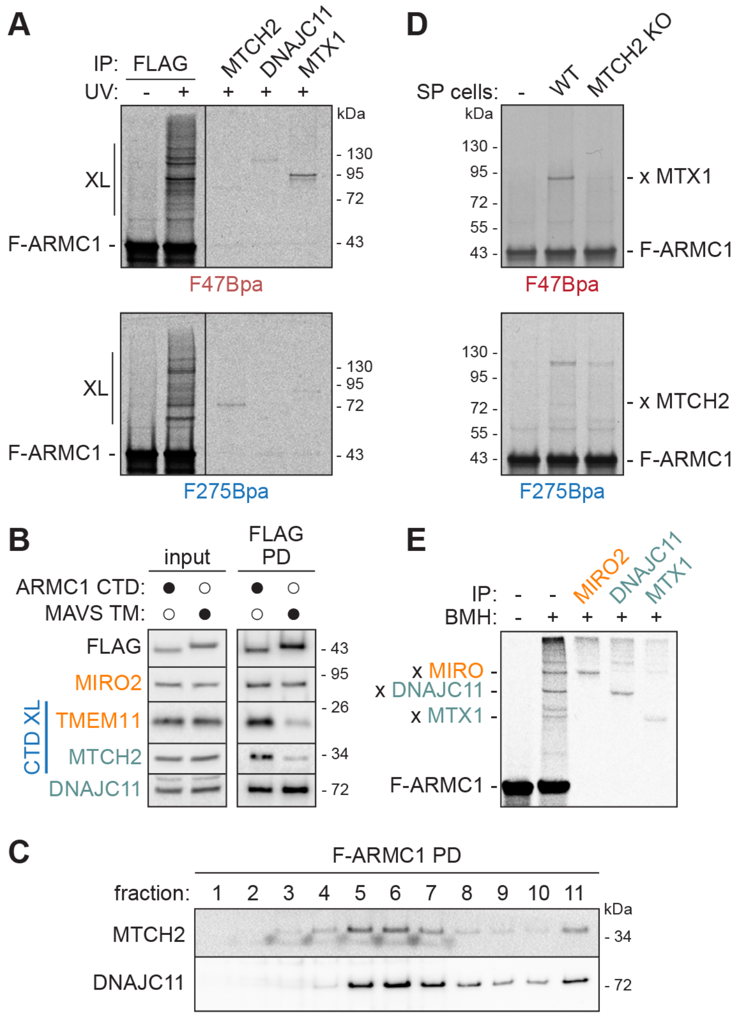

Fig. S5. Characterization of ARMC1 CTD receptors.

(A) Site-specific photocrosslinking reactions of radiolabeled F-ARMC1 with the UV-activated probe Bpa incorporated at position 47 in the helical domain (top) or at position 275 in the C-terminal domain (CTD, bottom) incubated with mammalian organelles were UV irradiated as indicated, immunoprecipitated (IP) for the indicated proteins, and assayed by SDS-PAGE and autoradiography. Note: MTX1 is the primary crosslinking partner with F-ARMC1(F47Bpa) and only weakly crosslinks to F-ARMC1(F275Bpa), while MTCH2 crosslinks strongly to F-ARMC1(F275Bpa) but not to F-ARMC1(F47Bpa).

(B) TMEM11 and MTCH2 are specific ARMC1 CTD receptors. ARMC1 knockout Flp-In 293 T-REx cells were complemented with F-ARMC1 containing an intact CTD, or an F-ARMC1 variant in which the CTD was replaced with the transmembrane helix (TM) of MAVS, a tail-anchored outer mitochondrial membrane protein. Solubilized membrane fractions (input) were subjected to F-ARMC1 variant pulldowns (PD) and analyzed by immunoblotting. Note: replacing the ARMC1 CTD with the MAVS TM impairs F-ARMC1 interaction with TMEM11 and MTCH2, but not with MIRO or DNAJC11.

(C) MTCH2 and DNAJC11 in F-ARMC1 affinity purifications co-migrate in size fractionations over a 10-30% sucrose gradient, assayed by SDS-PAGE and immunoblotting.

(D) MTX1 crosslinking to ARMC1 depend on MTCH2. Autoradiography of site-specific photocrosslinking reactions of radiolabeled F-ARMC1 with Bpa integrated as in (A) incubated with WT or MTCH2 knockout (KO) semi-permeabilized (SP) cells.

(E) Chemical crosslinking partners of ARMC1. Radiolabeled F-ARMC1 synthesized *in vitro* was incubated with semi-permeabilized cells, treated with the cysteine-reactive crosslinker bismaleimidohexane (BMH) as indicated, and subjected to immunoprecipitations (IP) to identify crosslinks to MIRO2, DNAJC11, and MTX1, analyzed by phosphorimaging.

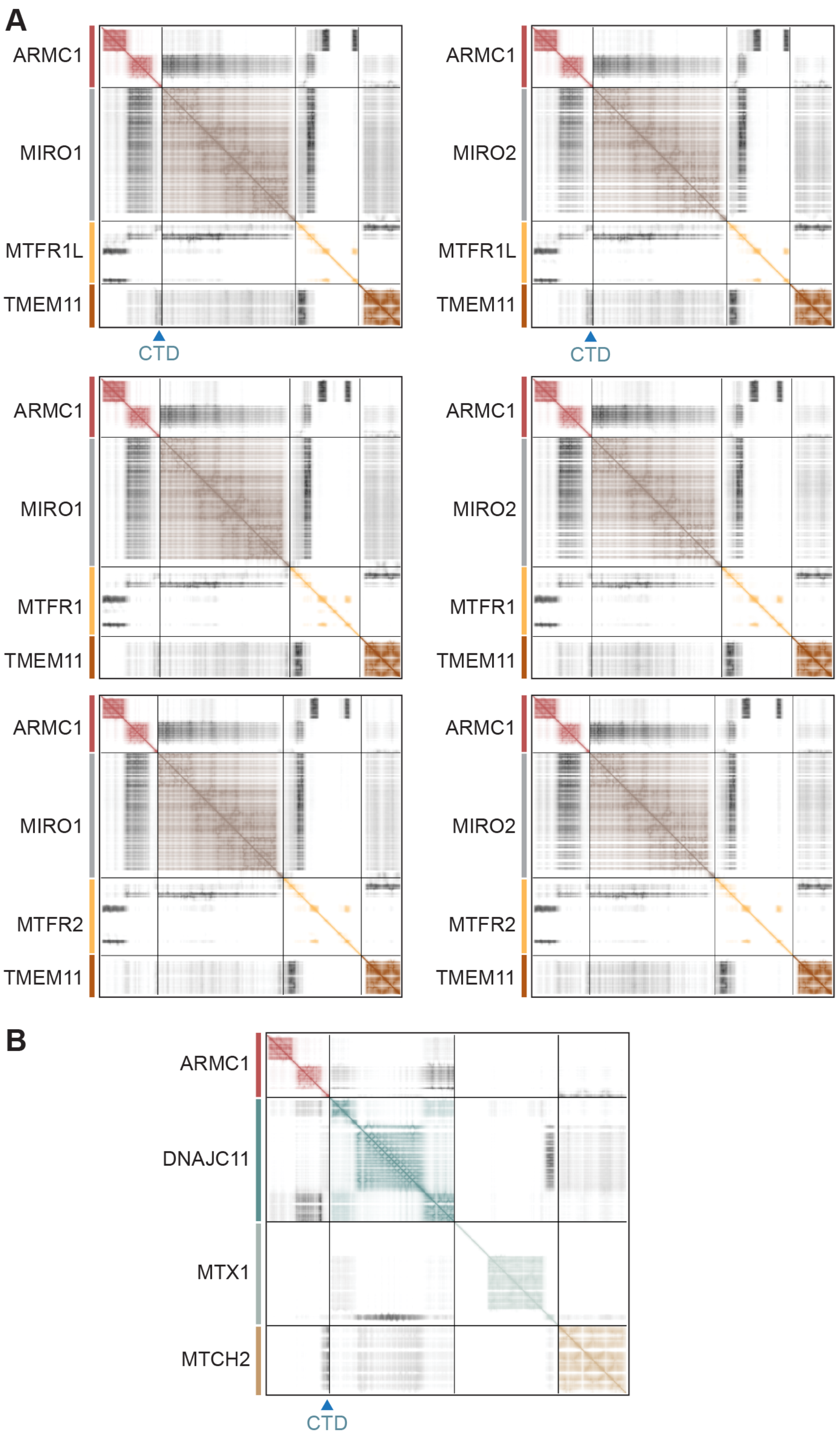

Fig. S6. Alphafold Predicted Aligned Error (PAE) plots.

Predicted aligned error (PAE) plots for Alphafold3 predictions of **(A)** ARMC1-MIRO-MTFR-TMEM11 complexes, with all combinations of MIRO and MTFR paralogs, and **(B)** the ARMC1-DNAJC11-MTX1-MTCH2 complex.

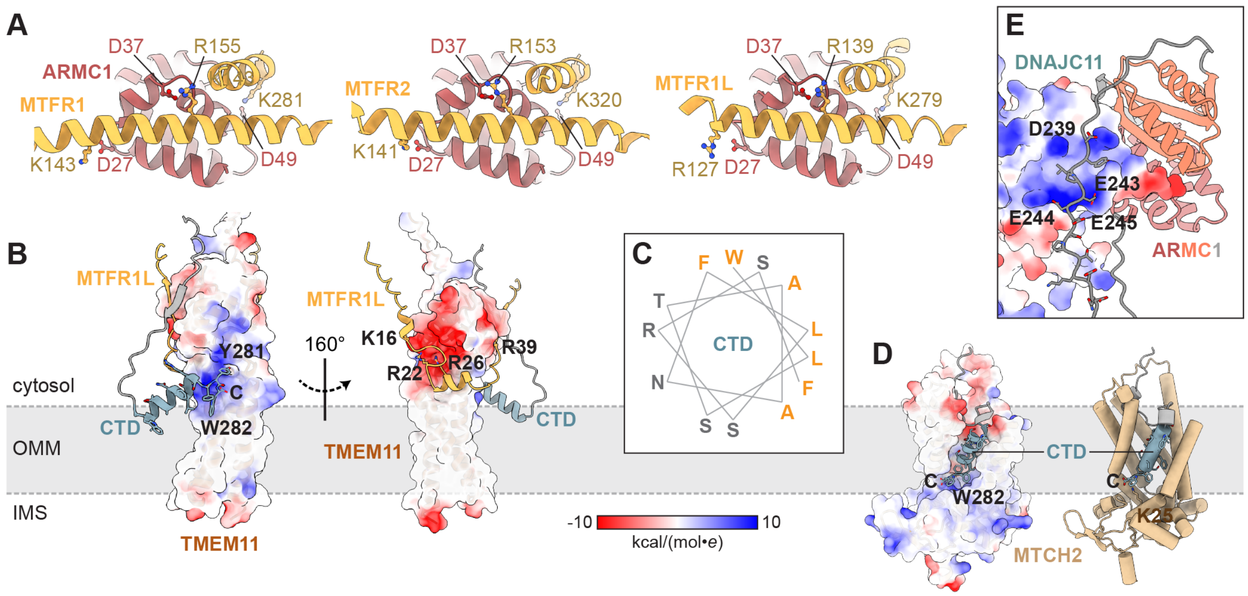

Fig. S7. ARMC1 interactions predicted by Alphafold.

(A) The predicted interaction interface between ARMC1 (dark red) and each MTFR (light orange) in Alphafold models. Key residues, including ARMC1 D37, are labeled.

(B) Predicted interaction interfaces between TMEM11 (colored by electrostatics), the ARMC1 C-terminal domain (CTD; light blue), and MTFR1L (light orange). Key ARMC1 CTD and MTFR1L residues that interact with basic and acidic patches on TMEM11 are indicated.

(C) The ARMC1 CTD forms an amphipathic helix. Helical wheel representation of residues 268-280 of the ARMC1 CTD. Hydrophobic amino acids are orange.

(D) The predicted interaction interface between the ARMC1 CTD (light blue) and MTCH2, colored by electrostatics (left) or tan (right). Note: putative interaction between the C-terminal carboxyl group of ARMC1 and a basic patch contributed by K25 of MTCH2.

(E) The predicted binding interface between ARMC1 (cartoon) and DNAJC11, colored by electrostatics. Note: interaction between acidic residues on ARMC1 (labeled and mutated in the 4K ARMC1 variant) with a basic patch on DNAJC11.

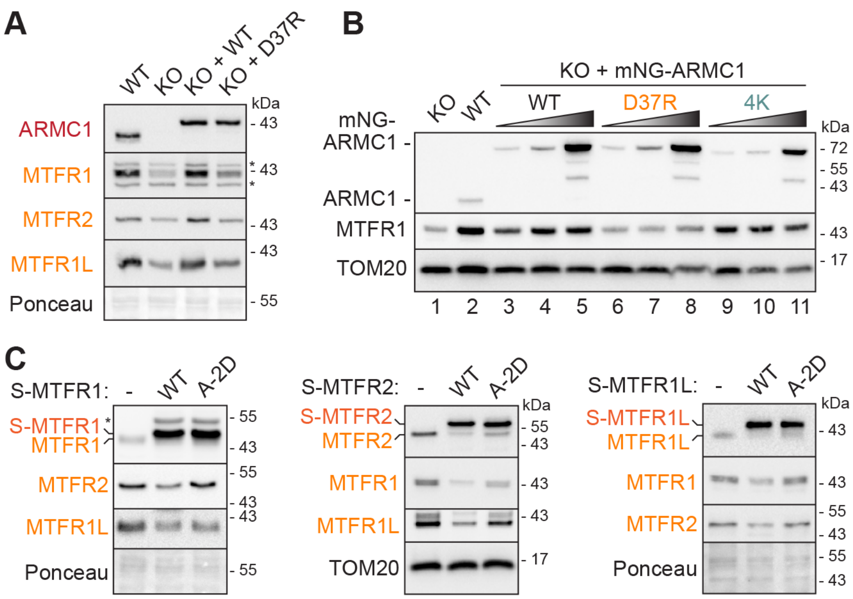

Fig. S8. MTFR stability requires interaction with ARMC1.

(A) Immunoblotting of lysates from wildtype (WT) or ARMC1 knockout (KO) Flp-In 293 T-REx cells without or with re-expression of WT or D37R F-ARMC1 predicted to disrupt the MTFR binding interface. Note: D37R F-ARMC1 does not rescue MTFR levels.

(B) Immunoblotting of lysates from WT or ARMC1 KO COS7 cells transduced with WT, D37R, or 4K mNeonGreen (mNG)-tagged ARMC1 and sorted by mNG levels (also used in Figs. 1A and 4A). Note: WT and 4K but not D37R mNG-ARMC1 rescues MTFR1 levels.

(C) Immunoblotting of the membrane fractions of Flp-In 293 T-REx cells stably overexpressing the indicated WT Strep-tagged MTFR (S-MTFR) or a variant (A-2D) containing two point mutations predicted to disrupt ARMC1 interaction. Note: destabilization of endogenous MTFRs upon overexpression of WT but not A-2D S-MTFR.

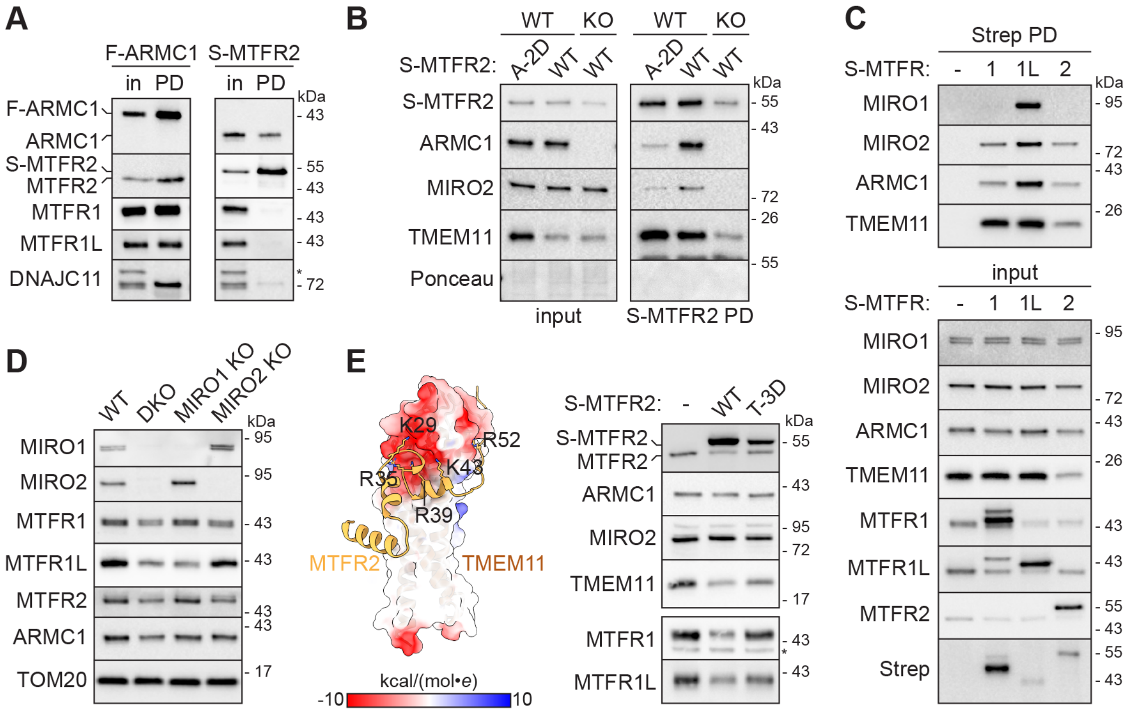

Fig. S9. Characterization of MTFR interactions.

(A) Inputs (in) and pulldowns (PD) of Flag-tagged ARMC1 (F-ARMC1, left) or Strep-tagged MTFR2 (S-MTFR2, right), assayed by SDS-PAGE and immunoblotting.

(B) Input or PD of wildtype (WT) or A-2D (R153D/K320D) S-MTFR2 expressed in WT or ARMC1 knockout (KO) cells, assayed by immunoblotting. Note: Mutating the ARMC1-binding interface (A-2D) of S-MTFR2 or knocking out ARMC1 impairs the PD of MIRO.

(C) Immunoblotting of the input (bottom) and PD (top) of Strep-tagged MTFR (S-MTFR) paralogs. Note: all three MTFRs pull down MIRO2 but only S-MTFR1L stably pulls down MIRO1.

(D) Immunoblotting of the membrane fractions from WT, MIRO1 KO, MIRO2 KO, and MIRO1 and MIRO2 double knockout (DKO) Flp-In 293 T-REx cells. Note: selective destabilization of MTFR1L in MIRO1 KO cells and of MTFR1 and MTFR2 in MIRO2 KO cells.

(E) Alphafold model of the TMEM11-MTFR2 interaction with TMEM11 colored by electrostatics (left). The MTFR2 residues involved in the interactions are labeled. Immunoblotting of the membrane fraction of cells expressing WT or K29D/R35D/R39D (T-3D) S-MTFR2 reveals that mutations at the MTFR-TMEM11 interface do not destabilize endogenous MTFRs or TMEM11 (right).

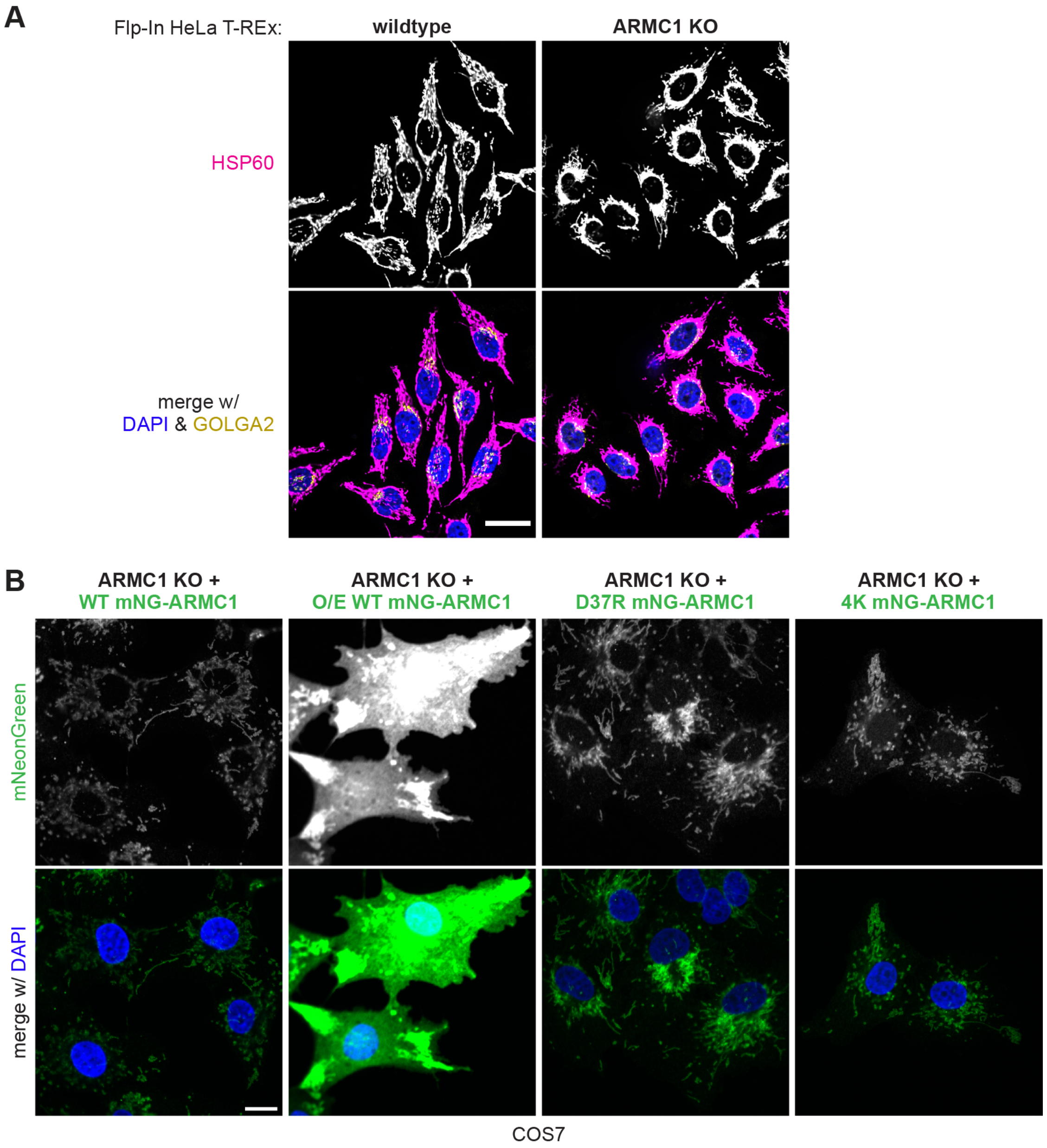

Fig. S10. Knocking out ARMC1 disrupts mitochondrial distribution.

(A) Immunofluorescence of wildtype and ARMC1 knockout (KO) Flp-In HeLa T-REx cells showing the mitochondrial protein HSP60 (top and magenta), the Golgi marker GOLGA2 (yellow), and nuclei (DAPI, blue). Scale bar, 30 µm.

(B) Immunofluorescence of ARMC1 KO COS7 cells complemented without or with the indicated mNeonGreen (mNG)-tagged ARMC1 variants, showing the mNeonGreen signal of mNG-ARMC1 variants (top and green) and nuclei (DAPI, blue). Scale bar, 15 µm. O/E, overexpressed.

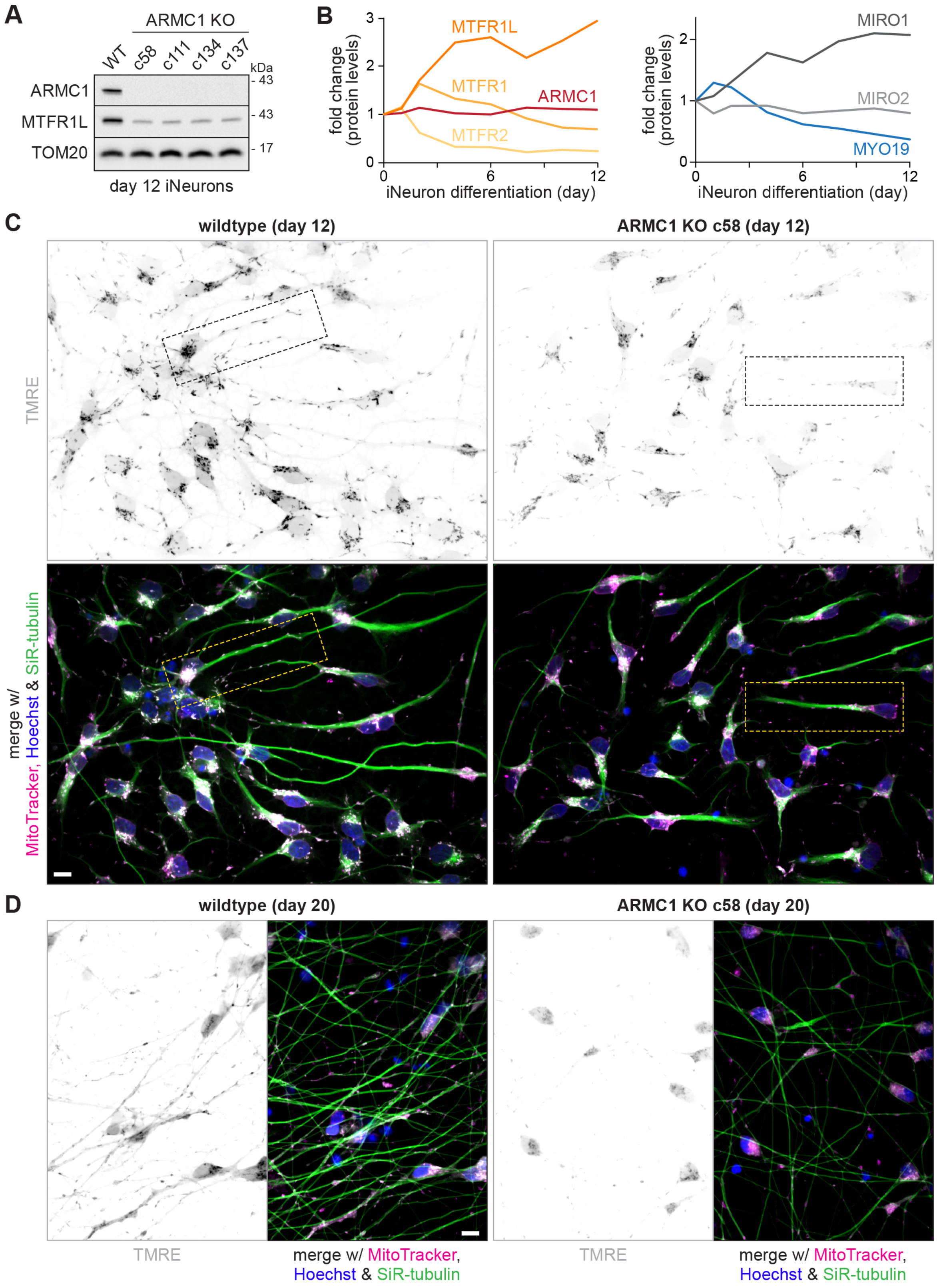
A
ARMC1 KO
WT
c58
c111
c134
c137
kDa
ARMC1
MTFR1L
TOM20
- 43
- 43
- 17
day 12 iNeurons
B
fold change (protein levels)
MTFR1L
MTFR1
ARMC1
MTFR2
MIRO1
MIRO2
MYO19
iNeuron differentiation (day)
C
wildtype (day 12)
ARMC1 KO c58 (day 12)
TMRE
merge w/ MitoTracker, Hoechst & SiR-tubulin
D
wildtype (day 20)
ARMC1 KO c58 (day 20)
TMRE
merge w/ MitoTracker, Hoechst & SiR-tubulin

Fig. S11. Consequences of knocking out ARMC1 in iNeurons.

(A) Immunoblotting of wildtype (WT) and four independent clones of ARMC1 knockout (KO) iNeurons after 12 days of NGN2-driven differentiation, showing destabilization of MTFR1L upon deleting ARMC1.

(B) Multiplexed proteomics over a timecourse of NGN2-driven iNeuron differentiation (ref. *49*) reveals that MTFR1L is specifically upregulated during neurogenesis, while the other MTFRs are downregulated (left), and that MIRO1 is also upregulated (right).

(C) Live-cell images of wildtype or ARMC1 KO iNeurons after 12 days of NGN2-induced differentiation showing TMRE staining of polarized mitochondria (top; gray), MitoTrackerGreen staining of total mitochondria, SiR-Tubulin staining, and Hoechst staining of nuclei (merge, bottom). Insets shown in Fig. 4D are boxed in yellow. Scale bar, 10 μm.

(D) Live-cell images of wildtype or ARMC1 KO iNeurons after 20 days of differentiation showing TMRE staining (left; gray) and merged channels (right) as in (C).

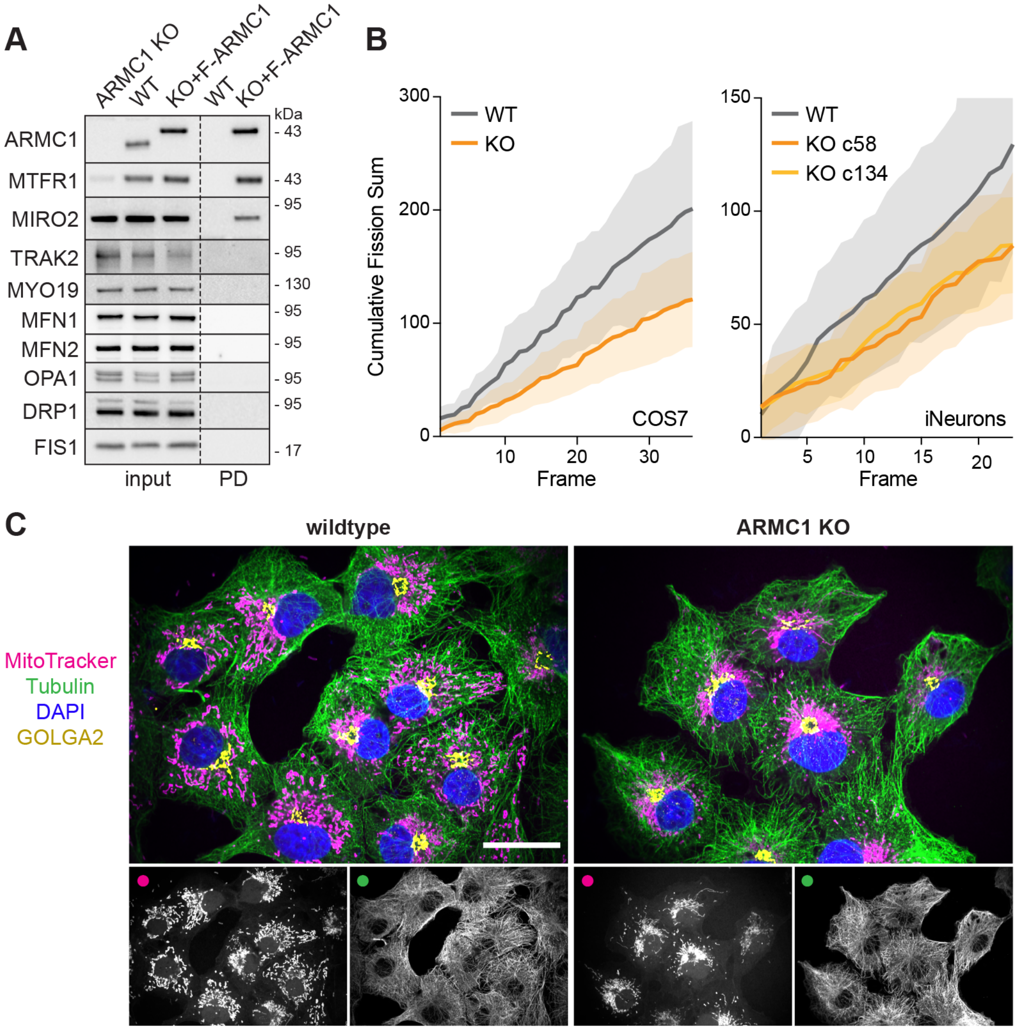

Fig. S12. Consequences of ARMC1 deletion on mitochondrial morphology.

(A) Immunoblotting of F-ARMC1 pulldowns (PD) and input membranes from wildtype (WT) or F-ARMC1 rescue cells show no copurification of the indicated fission, fusion, or cytoskeletal factors besides MIRO and MTFR.

(B) Cumulative mitochondrial fission events observed by live-cell imaging of MitoTracker in WT (n=105) or ARMC1 KO (n=56) COS7 cells and WT (n=363) or ARMC1 KO iNeurons [c58 (n=149) and c134 (n=108) refer to different ARMC1 KO clonal lines] after 12 days of differentiation. Each frame is 7.5 sec.

(C) Immunofluorescence of wildtype and ARMC1 KO COS7 cells showing no differences in the microtubule (Tubulin alpha, green) network. Scale bar, 30 μm.

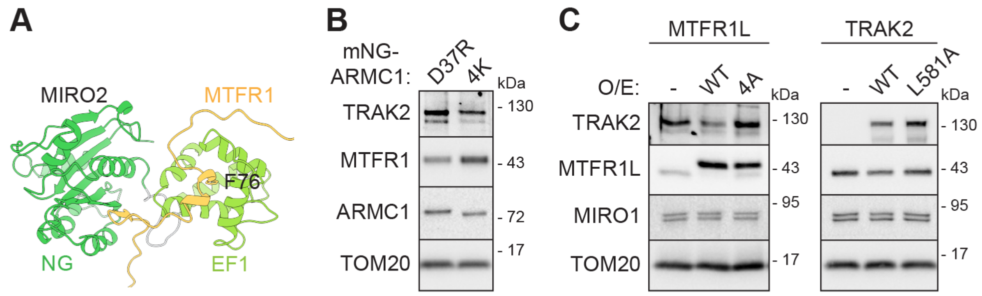

Fig. S13. ARMC1-mediated MIRO-MTFR assembly competes with TRAK recruitment.

(A) Alphafold model of MTFR1 with MIRO2 predicts a common interface on MIRO that also interacts with other MTFRs and TRAK.

(B) MIRO-MTFR assemblies compete with TRAK recruitment. Immunoblotting of membrane fractions from ARMC1 knockout COS7 cells re-expressing mNeonGreen (mNG)-tagged D37R or 4K ARMC1. Note: increased membrane association of TRAK2 with D37R ARMC1.

(C) Immunoblotting of membrane fractions of Flp-In 293 T-REx cells without or with overexpression (O/E) of wildtype (WT) or 4A (L62A/D64A/I65A) MTFR1L (left), or of WT or L581A TRAK2 (right). Note: WT but not 4A MTFR1L O/E reduces TRAK2 membrane association, and WT but not L581A TRAK2 O/E reduces MTFR1L membrane association.

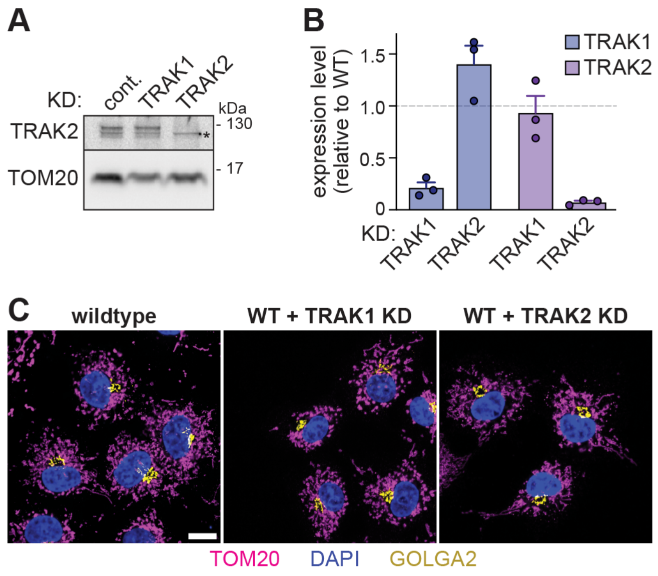

Fig. S14. Consequences of depleting TRAK.

(A) Immunoblotting of COS7 cells treated with control (cont.) siRNAs or siRNAs to knock down (KD) TRAK1 or TRAK2.

(B) Knockdown efficiencies of TRAK1 and TRAK2 siRNAs assayed by qPCR. Shown are mean + s.e.m. of 3 biological replicates, each with 3-4 technical replicates. The individual means are indicated.

(C) Immunofluorescence of wildtype COS7 cells without or with siRNA-mediated KD of TRAK1 or TRAK2 showing the mitochondrial protein TOM20 (magenta), the Golgi marker GOLGA2 (yellow), and nuclei (DAPI, blue). Scale bar, 15 µm.

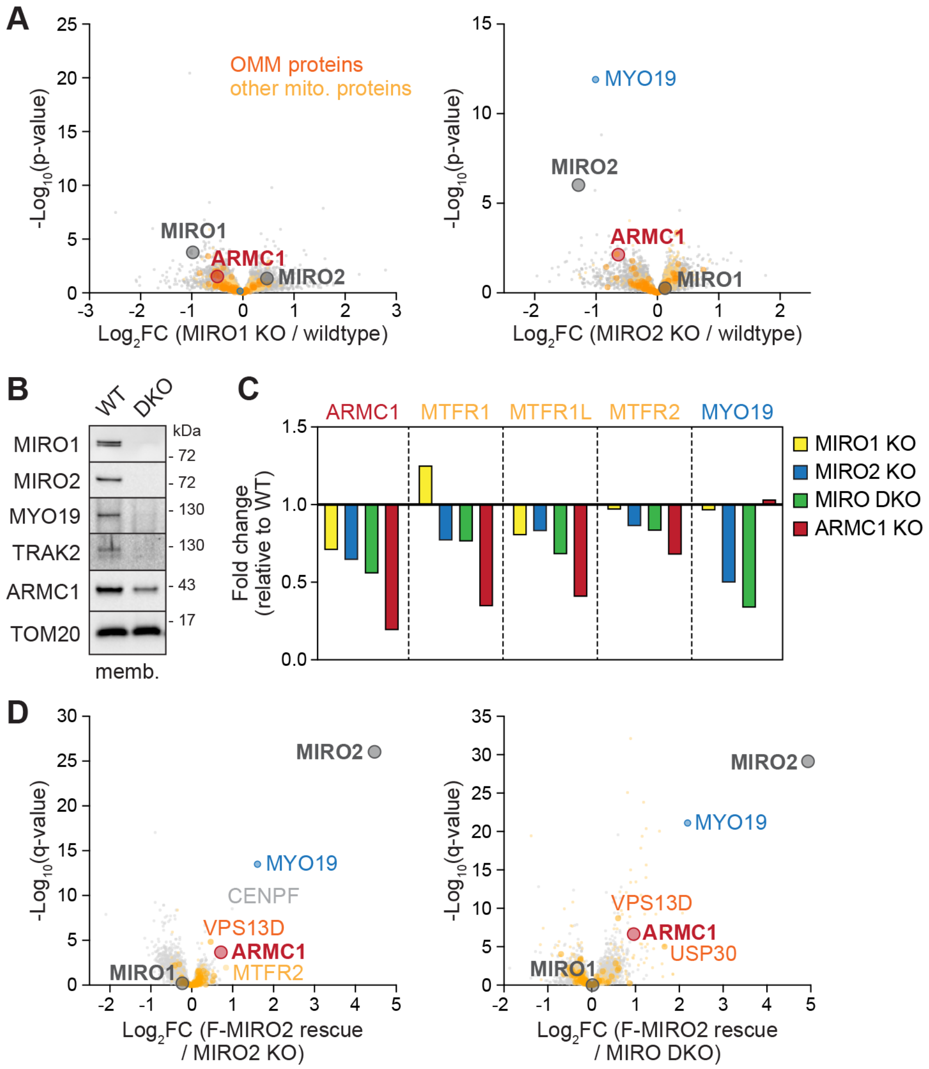

Fig. S15. Impact of MIRO on the cellular membrane fraction proteome.

(A) Volcano plot of multiplexed proteomics data showing the fold-change (FC) of proteins associated with cellular membranes in MIRO1 (left) or MIRO2 (right) knockout (KO) Flp-In 293 T-REx cells relative to wildtype cells. Individual, outer mitochondrial membrane (OMM), and other mitochondrial (mito.) proteins are indicated. Note: depletion of membrane-associated ARMC1 upon knocking out MIRO1 or MIRO2.

(B) Immunoblotting of cellular membranes (memb.) from wildtype (WT) or MIRO1 and MIRO2 double knockout (DKO) Flp-In 293 T-REx cells.

(C) Fold-change in the membrane-associated levels of the indicated proteins (top) in the KO or DKO cell line indicated by colored bars relative to wildtype (WT) cells from multiplexed proteomics data as in (A). Note: ARMC1 levels decrease upon deletion of either MIRO1 or MIRO2 (first block); only ARMC1 and MTFR1L levels decrease in MIRO1 KO cells (yellow bars); MYO19 levels only decrease in the absence of MIRO2 (last block).

(D) Volcano plot showing the fold-change (FC) of proteins associated with cellular membranes in MIRO2 KO (left) or MIRO DKO (right) cells with or without re-expression of Flag-tagged MIRO2 (F-MIRO2).

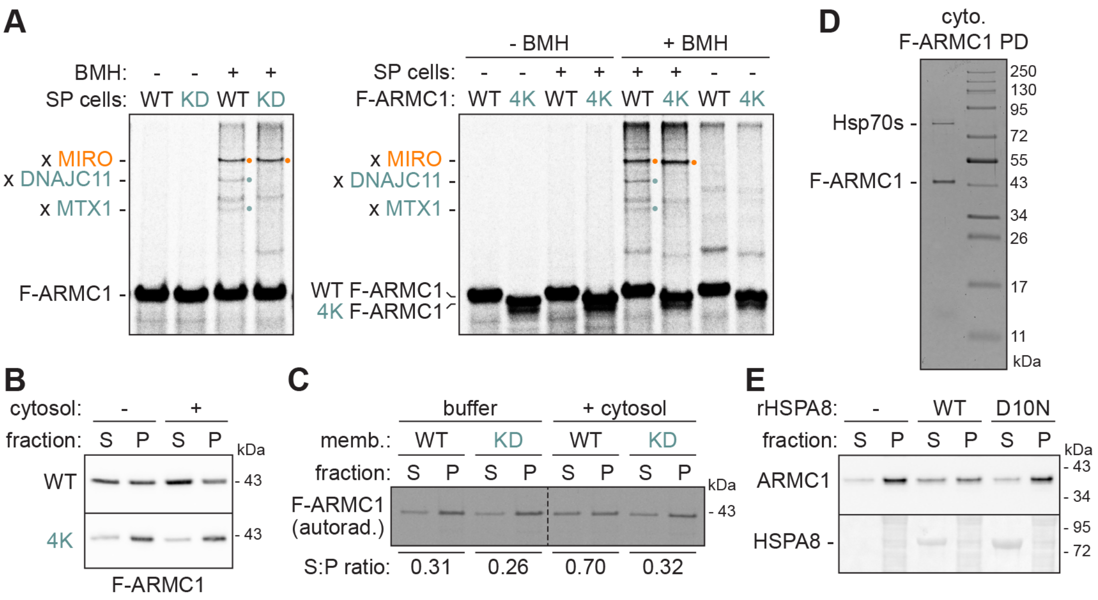

Fig. S16. DNAJC11 mediates ARMC1 release from mitochondria.

(A) Crosslinking reactions, using bismaleimidohexane (BMH) as indicated, of radiolabeled wildtype (WT) or 4K F-ARMC1 incubated with semi-permeabilized (SP) cells without or with siRNA-mediated knockdown (KD) of DNAJC11. Crosslinks to MIRO, DNAJC11, and MTX1 are indicated. Note: unlike WT F-ARMC1, 4K F-ARMC1 does not crosslink to DNAJC11 or MTX1.

(B) Flag immunoblots of F-ARMC1 mitochondrial release assays, in which the membrane fractions of WT or 4K F-ARMC1 rescue cells were incubated without or with cytosol and then separated into supernatant (S) and membrane pellet (P) fractions as in Fig. 6E. Note: 4K F-ARMC1 unable to interact with DNAJC11 does not release from mitochondria.

(C) Autoradiography of radiolabeled F-ARMC1 release assays, in which *in vitro* translations of radiolabeled F-ARMC1 were first incubated with the membrane fraction (memb.) of cells without or with DNAJC11 KD, which were then reisolated for release assays as in Fig. 6E. The ratios of radiolabeled F-ARMC1 signal in the supernatant versus the pellet (S:P) for each condition is shown below and, in Fig. 6F, for independent replicates. Note: less F-ARMC1 is released into the supernatant when DNAJC11 is depleted.

(D) SDS-PAGE and Coomassie staining of F-ARMC1 affinity purifications from the cytosolic (cyto.) fraction of Expi293F cells. Note: Hsp70s are the primary proteins copurified.

(E) Immunoblotting for endogenous ARMC1 in mitochondrial release assays without or with WT HSPA8 (rHSPA8), a cytosolic Hsp70, or the D10N variant that is catalytically impaired. Note: WT but not D10N rHSPA8 facilitates ARMC1 release.

Table S1. (separate file)
TMT-MS values of WT, ARMC1 KO, and F-ARMC1 rescue Flp-In 293 T-REx cells.

Table S2. (separate file)
TMT-MS values of WT, ARMC1 KO, and F-ARMC1 rescue Hela T-REx cells.

Table S3. (separate file)
Protein identifications of F-ARMC1 affinity purification gel bands.

Table S4. (separate file)
TMT-MS values of membrane-associated proteins from Flp-In 293 T-REx cells with or without MIRO or ARMC1 deletion.

Table S5. (separate file)
TMT-MS values of membrane-associated proteins from Flp-In 293 T-REx cells with MIRO deletion and F-MIRO2 rescue.

Movie S1. (separate file)
Two examples of mitochondrial fission events (denoted by red arrows) in COS7 cells. Scale bar, 5 μm.
